# Risk stratification for significant acute traumatic intracranial hemorrhage in older adults after a ground-level fall: A prospective multicentre cohort study

**DOI:** 10.1371/journal.pmed.1004935

**Published:** 2026-08-10

**Authors:** Xavier Dubucs, Cassandre Even, Jemery Guenezan, Margaux Lagache, Barbara Villoing, Lorraine Pouzet, Marián Sedlák, Radoslav Morochovič, Yann-Érick Claessens, Frédéric Balen, Valérie Boucher, Sandrine Charpentier, Éric Mercier, Marcel Émond

**Affiliations:** 1 Emergency Department, Toulouse University Hospital Centre, Toulouse, France; 2 Centre d’Epidémiologie et de Recherche en santé des Populations, INSERM 1295 Université de Toulouse, Toulouse, France; 3 Research Center of Laval University, Axe santé des populations et pratiques optimales en santé, Québec, Québec, Canada; 4 Emergency Department, Poitiers University Hospital Centre, Poitiers, France; 5 Emergency Department, Cochin University Hospital Centre, Paris, France; 6 Department of Trauma Surgery, Faculty of Medicine, Pavol Jozef Safarik University, Kosice, Slovakia; 7 Louis Pasteur University Hospital, Kosice, Slovakia; 8 Emergency Department, Princesse Grace Hospital Center, Monte Carlo, Monaco; Uniformed Services University: Uniformed Services University of the Health Sciences, UNITED STATES OF AMERICA

## Abstract

**Background:**

Falls among older adults are now the leading cause of traumatic brain injury worldwide. We aimed to identify historical and clinical characteristics including the visible head impact location indicative of significant acute traumatic intracranial hemorrhage in older patients presenting to emergency department with mild traumatic brain injury subsequent to a ground-level fall.

**Methods and findings:**

We conducted a multicentre prospective cohort study across five university-affiliated emergency departments over a 2-year period (1 July 2023 to 30 June 2025) in Europe. We included patients aged 65 years or older who presented with mild traumatic brain injury (defined as head trauma with a Glasgow Coma Scale score of 13–15 upon emergency department presentation) following a ground-level fall and who underwent a computed tomography scan. The primary outcome was significant acute traumatic intracranial hemorrhage, defined as a neuroimaging radiological interpretation system (NIRIS) score > 1. Predictors were identified using logistic regression and recursive partitioning. A predictor was included in the decision rule if its association with the primary outcome and its interobserver reliability were strong. Using logistic regression, associations between independent variables and the outcome were adjusted for age, antithrombotic medication, and precipitating factors for the fall. Internal validation was performed using bootstrapping. The study included 1,620 patients (mean age, 84.6 ± 8.5 years). A significant acute traumatic intracranial hemorrhage was identified in 72 patients (4.4%, 95% CI [3,6]) of which five (0.3%, 95 CI% [0,1]) required urgent neurosurgical intervention. Eight criteria were identified as strong and reliable predictors: visible forehead-scalp impact, Glasgow Coma Scale score below baseline, focal neurological deficit, sign of basal skull fracture, acute confusion, vomiting, loss of consciousness, and headache. We then derived two clinical decision rules (PIWI 1 and PIWI 2), which both showed 100% sensitivity (95% CI [95,100]) with specificities ranging from 25.3% (95% CI [23,28]) to 43.6% (95% CI [41,46]). Application of either clinical decision rule would have allowed reductions (41.7% or 24.2%) of the numbers of patients sent to the CT scan unit. Internal validation confirmed the strong performance of both rules, based on C-statistics of 0.84 (95% CI [0.8,0.9]) and 0.79 (95% CI [0.7,0.9]). Because we only included patients who underwent a head CT scan during their emergency department stay, the potential for selection bias must be considered. Additionally, a risk of misclassification bias exists because we did not perform a centralized independent review of the CT scans.

**Conclusion:**

Our findings revealed that factors drawn from patient history and physical examination were associated with significant acute traumatic intracranial hemorrhage in older adults after a ground-level fall. Incorporating these factors into decision rules could provide a reliable strategy to stratify risk and reduce unnecessary CT scan. We hypothesize that the PIWI 1 rule could be used in patients with a clear history of the fall, while the PIWI 2 rule could be applied in other cases. Such rules need to be validated externally and independently for their implementation in clinical practice, but may already be of aid for identifying high-risk patients.

## Author summary

**Why was this study done?:**

- Non-contrast computed tomography scan of the head is widely recommended for older patients who have sustained a ground-level fall, to rule out traumatic intracranial hemorrhage.
- Recent epidemiological studies indicate that 95% of scans of such patients show no hemorrhaging.

**What did the researchers do and find?:**

- We conducted a multicentre prospective cohort study across five university-affiliated emergency departments over a 2-year period (1 July 2023 to 30 June 2025) in Europe.
- Two highly sensitive decision rules, both based on simple and reliable criteria, have been designed specifically for application to this patient population.
- Internal validation suggests high performance for both rules within the cohort.

**What do these findings mean?:**

- Our study provides a novel approach that takes visible head impact location into consideration to reduce the use of computed tomography in this patient population.
- To apply the first rule (PIWI 1), a clear history of falls is required, while the second (PIWI 2) could be used in other cases.
- Application of either rule could reduce use of computed tomography (41.7% or 24.2%) without missing any significant acute traumatic intracranial hemorrhage.
- Because we only included emergency department patients who underwent a head scan, and did not have those scans double-checked by a central team, there is a risk our results could be skewed or misclassified
- Before being proposed for implementation in clinical practice, these rules need to be validated in other healthcare settings using large cohorts with a large number of outcome events.

## Abstract 3

Using data from a prospective cohort study, Xavier Dubucs and colleagues design clinical decision rules to guide use of CT scans in older patients after a fall and at risk of brain hemorrhage.

## Introduction

The global epidemiology of traumatic brain injury has evolved rapidly in recent years [1]. Traumatic brain injury stands out as the most prevalent neurological disorder worldwide, with an annual incidence of approximately 60 million new cases, making it a major public health and economic challenge [2,3]. High-income countries have experienced a marked rise in traumatic brain injury incidence among older adults [4], the mean age of patients with traumatic brain injuries having nearly doubled in recent years. The mechanisms of injury have also changed, with falls now surpassing traffic accidents as the leading cause in these countries [5], where 20%–30% of individuals aged 65 and older and up to 50% of those over 80 experience at least one fall annually [6]. Among older adults presenting to an emergency department after a fall, the most frequent traumatic injury is to the brain, representing 25%–42% of cases [7,8]. These trends are placing significant strain on healthcare systems. Recent epidemiological data indicate that traumatic brain injury-related ED visits among older patients have increased disproportionately, with some European countries reporting a 244% rise over the past decade [9].

Non-contrast computed tomography (CT) of the head remains the standard reference for evaluating traumatic brain injury to exclude acute intracranial hemorrhage. Current clinical decision rules such as the Canadian CT Head Rule (for ≥65-year-olds) and the New Orleans Criteria (for ≥60-year-olds) consistently identify older age as a risk factor for intracranial hemorrhage [10,11]. Both were developed over two decades ago and at that time relied primarily on data from younger adults (mean ages of 36 and 38.7 years, respectively). Some researchers have proposed adapting these rules by removing the age threshold, but this resulted in suboptimal performance [12]. In addition, most of these clinical decision rules exclude patients on antithrombotic medication, although this group represents a growing proportion of the aging population [13]. Ground-level fall is now the reason for 40% of CT head scans performed on patients with mild head injury [14]. While emerging evidence indicates that 90%–95% of these scans yield normal findings [15,16]. Concerns with exposure to radiation and the economic burden on healthcare systems appear to warrant more selective use of CT imaging in older adults [17].

Our preliminary studies of this patient population suggested that clinical findings such as the specific location of visible head impact could be of aid in assessing the likelihood of traumatic intracranial hemorrhage [18–20]. To date, no clinical decision rule incorporating visible head impact location has been developed for such assessment in cases of ground-level fall. The Computed Tomography of the Head for Patients at Advanced Age (CTHEAD) rule, derived retrospectively then validated prospectively, included all trauma mechanisms (ground-level fall in 70% of cases) while excluding patients on anticoagulant therapy [21]. The Falls Decision Rule was derived prospectively and recently validated for all older ground-level-fall patients regardless of head trauma status [22,23]. The Florida Geriatric Head Trauma CT Clinical Decision Rule was derived and validated by proposing a rule based on clinical findings and the mode of patient arrival at the emergency department [24].

In this study, we aimed to derive a set of criteria integrating the visible head impact location to assess the likelihood of significant acute traumatic intracranial hemorrhage and identifying cases of ground-level-fall-associated traumatic brain injury that do not require a head CT scan.

## Methods

### Study design

This multicentre prospective cohort study was conducted in five university-affiliated emergency departments located in France, Monaco, and Slovakia, with a combined annual emergency department attendance of approximately 352,000 visits. These centers were selected due to their involvement in a preliminary study on this topic [20]. An observational design was chosen, since it aligns with the current stage of development for clinical decision rules [25]. This study was reported as per the Transparent Reporting of a Multivariable Prediction Model for Individual Prognosis Or Diagnosis (TRIPOD) statement (S2 Checklist TRIPOD) [26]. This study is reported as per the Strengthening the Reporting of Observational Studies in Epidemiology (STROBE) guideline (S1 Checklist STROBE) [27].

### Participants

Patients were eligible for inclusion if aged at least 65 years and presenting to an emergency department with mild traumatic brain injury due to a ground-level fall occurring up to 24 hours prior to arrival, and having undergone a head CT scan. The age threshold of 65 years was selected based on existing clinical decision rules, which consistently identify this age as a risk factor for intracranial lesions [10,11]. Mild traumatic brain injury was defined as head trauma with a Glasgow Coma Scale score between 13 and 15 upon presentation, and was presumed present if the patient or a witness reported a head impact in conjunction with the fall, and/or if any external sign of head trauma was noted by the emergency clinical physician during examination. Transient neurological symptoms, such as loss of consciousness, were not required to define mild traumatic brain injury. Our preliminary studies of this population have shown that nearly 30% of these patients exhibit cognitive impairment, making it difficult to identify transient neurological symptoms [18,19,28]. Ground-level falls were defined as any fall from a standing position, off a chair, or out of bed. High-kinetic trauma, including collision with motorized vehicles or bicycles, were excluded from the study. We also excluded patients with hemophilia (congenital or acquired) and those with primary hemostasis disorders (e.g., von Willebrand disease, immune thrombocytopenic purpura). Patients on antithrombotic therapy (antiplatelet or anticoagulant agents) were not excluded. Participants provided oral informed consent to participate in the study and received an information sheet explaining the research. In cases of cognitive impairment or altered neurological status, consent was obtained from the patient’s designated representative.

### Data collection

Consecutive patients were assessed by emergency physicians. The decision to perform a CT scan of the head was left to the judgement of the treating physician. Our preliminary study indicated that CT scans are performed in 75.2% of such cases [28].

Enrollment was conducted by the senior emergency physician, who completed a standardized data collection (upon patient presentation in the emergency department) before ordering the CT scan. Potential predictors were therefore recorded with blinding to the outcome [25]. Physicians were familiarized with the study and the standardized data collection process through a one-hour online session with the investigators, who had themselves been trained by the study’s principal investigators in a similar session. Among patients with significant acute traumatic intracranial hemorrhage, death or neurosurgical intervention (occurring up to 7 days after emergency department arrival) was noted on site from patient medical records.

Potential risk factors for intracranial hemorrhage were reported based on patient history and physical examination. For each potential predictor related to the fall history, a “not assessable” option was available for selection. Our preliminary studies have shown that obtaining a reliable and exact history of the trauma is not possible in nearly 30% of this population [19]. Wherever feasible, we asked the initial attending physician to have a second on-duty emergency physician independently complete a second data collection form, blinded to the initial collection, to calculate interobserver agreement. The precipitating factors of the falls were reported based on the clinical history and any ancillary tests performed in the emergency department, including environmental causes, syncope, dizziness, and alcohol intoxication. These ancillary tests were ordered at the discretion of the treating physician. Alcohol intoxication was determined based on patient or witness history, or suggestive findings on physical examination, such as alcohol odor on the breath. “Not assessable” was recorded if the available information in the emergency department did not allow specification of the factors that precipitated the fall. Symptoms presented by the patient after the fall were documented if identified during the patient interview or observed upon admission to the emergency department, including loss of consciousness, fall-related amnesia (retrograde or anterograde), acute confusion, or seizure. Headache was defined as persistent, diffuse head pain following the fall. Localized pain at the site of a visible head impact was not considered sufficient to meet this definition. Vomiting was defined as any emesis occurring after the traumatic event. Glasgow Coma Scale scores below the baseline were noted upon emergency department presentation, along with de novo pupil abnormalities, focal neurological deficits, and signs of basal skull fracture (raccoon eyes, otorrhea, otorrhagia, rhinorrhea). The baseline Glasgow Coma Scale score was presumed from the patient’s pre-injury status, based on interviews with the patient or witnesses. Visible head impact characteristics were categorized as bruise, subcutaneous hematoma, or wound requiring sutures. Multiple characteristics could be identified for a single impact. Regarding visible head impact location, the emergency physician had to select only one location: no visible impact, facial impact, forehead impact, or scalp impact. Facial impact location included the entire face, including the eyebrows. The scalp was defined as the hair-bearing area, and the forehead region was the area between the eyebrows and the scalp (above the eyebrows and below the hairline). In cases of multiple impacts, the physician had to select the primary impact associated with the head trauma. Frailty was assessed by the emergency treating physician using the Clinical Frailty Scale [29]. Patients were then categorized as robust (1–3), frail (4–6), severely frail (7–9) based on this scale. Other traumas associated with mild traumatic brain injuries were reported after discharge from the emergency department, based on the emergency medical records kept by a research assistant at each center or by the site investigator.

### Outcome measures

The primary outcome was significant acute traumatic intracranial hemorrhage, based on CT scan. The Neuroimaging Radiological Interpretation System (NIRIS) was used to define this outcome [30]. This classification system provides a standardized approach for interpreting imaging findings in patients with traumatic brain injury, and has demonstrated and validated predictive value for patient outcomes and management [31,32]. According to this classification, significant acute traumatic intracranial hemorrhage was defined as any intracranial hemorrhage with a NIRIS score greater than 1 (epidural hematoma, subdural hematoma, parenchymal hematoma, or parenchymal contusion > 0.5 mL; mild or moderate hydrocephalus; midline shift; intraventricular hemorrhage; diffuse axonal injury). Consistent with recent literature, a single head CT scan is sufficient in this population to rule out acute traumatic hemorrhage, even in patients on antithrombotic medication [33]. All CT scan results were reviewed by local attending neuroradiologists, who were blinded to the contents of the data collection forms but had access to the usual clinical information. Based on the neuroradiologist’s report, the findings were recorded in the REDCap platform by the site investigator, blinded to the potential predictors.

Secondary outcomes included any neurosurgical intervention and in-hospital traumatic brain injury-related death occurring within seven days of emergency department presentation.

### Statistical analysis

We developed a clinical decision rule whereby significant acute traumatic intracranial hemorrhage was presumed excludable in the absence of any risk factors. If one or more risk factors were present, the rule recommended performing a CT scan. This rule included the minimal number of predictor variables required to achieve 100% sensitivity. First, bivariate analyses were conducted to assess the strength of association between each potential predictor and the primary outcome, using Chi-square or Fisher’s exact test for nominal variables and Mann-Whitney or Wilcoxon *t* test for continuous variables, as appropriate. Predictors included in the rule were then identified through logistic regression and recursive partitioning. In line with standard methodology, combining these two techniques allows for correct patient classification regarding the outcome while minimizing the number of predictors. Different combinations of the visible head impact variable were tested during these analyses (e.g., scalp impact versus other; forehead-scalp impact versus other). A stepwise backward selection process, based on *p*-values and the Akaike Information Criterion (AIC), was used to refine the predictive factors in the final regression model. Variables with a *p*-value < 0.25 in logistic regression were further evaluated using recursive partitioning analysis. The interobserver agreement for each variable was assessed using the *κ* coefficient or Spearman’s interclass correlation coefficient. A predictor was included in the rule only if it was both strongly associated with the primary outcome and displayed good interobserver reliability (*κ* > 0.6). The reliability of each clinical decision rule was evaluated by measuring the interobserver agreement for all predictors included in the rule. Our goal was to develop a clinically relevant, reliable, and easily implementable decision rule for routine practice.

The prediction model was assessed first on apparent performance. The C-statistic was calculated to measure model discrimination ability. Internal validation was performed using bootstrapping [34]. Five hundred bootstrap samples were generated to assess performance of the final model. The *C*-Statistic was also calculated to estimate the area under the receiver operating characteristics curve of the final model on the bootstrap samples. The calibration of the final model was assessed by estimating calibration-in-the-large and the calibration slope [34]. The calibration plot was generated to illustrate the agreement between observed and expected risks of significant acute traumatic intracranial hemorrhage, with patients grouped by deciles of predicted risk.

No imputation was performed for missing data. The number of patients was reported for each variable, and statistical analyses were conducted using complete-case analysis. All analyses were performed using STATA BE 19.5, and bootstrap validation was carried out using the *bsvalidation* command [35].

### Sample size

The sample size was based on a preliminary study in which five binary predictors (Glasgow Coma Scale score below baseline, amnesia, loss of consciousness, vomiting, and subcutaneous hematoma) and one predictor with three categories (visible head impact location: no impact, facial impact, fronto-scalp impact) were identified [18]. These predictors were confirmed also through a systematic review and meta-analysis in this population. [16] Consistent with recent literature, we assumed a 5% prevalence of significant acute traumatic intracranial hemorrhage in this population [15]. Following the widely adopted rule of requiring at least 10 events per variable category, the target sample size for this study was 1,600 patients, which was estimated to yield 80 patients with significant acute traumatic intracranial hemorrhage [36].

### Ethical approval

Patients were informed that their anonymized data would be used for research, as required for approval by French research ethics authorities. The data protection officer validated the study and ensured that it met all necessary criteria, including the General Data Protection Regulation (GDPR). The institutional scientific board of Toulouse University Hospital’s Department of Clinical Research and Innovation approved this protocol. It was registered in the Toulouse University Hospital data study registry (registration number: RnIPH 2023-54) and was covered under the MR-004 reference methodology (CNIL number: 2206723 v 0) (the study protocol is available in the S1 File).

## Results

**Fig 1:**
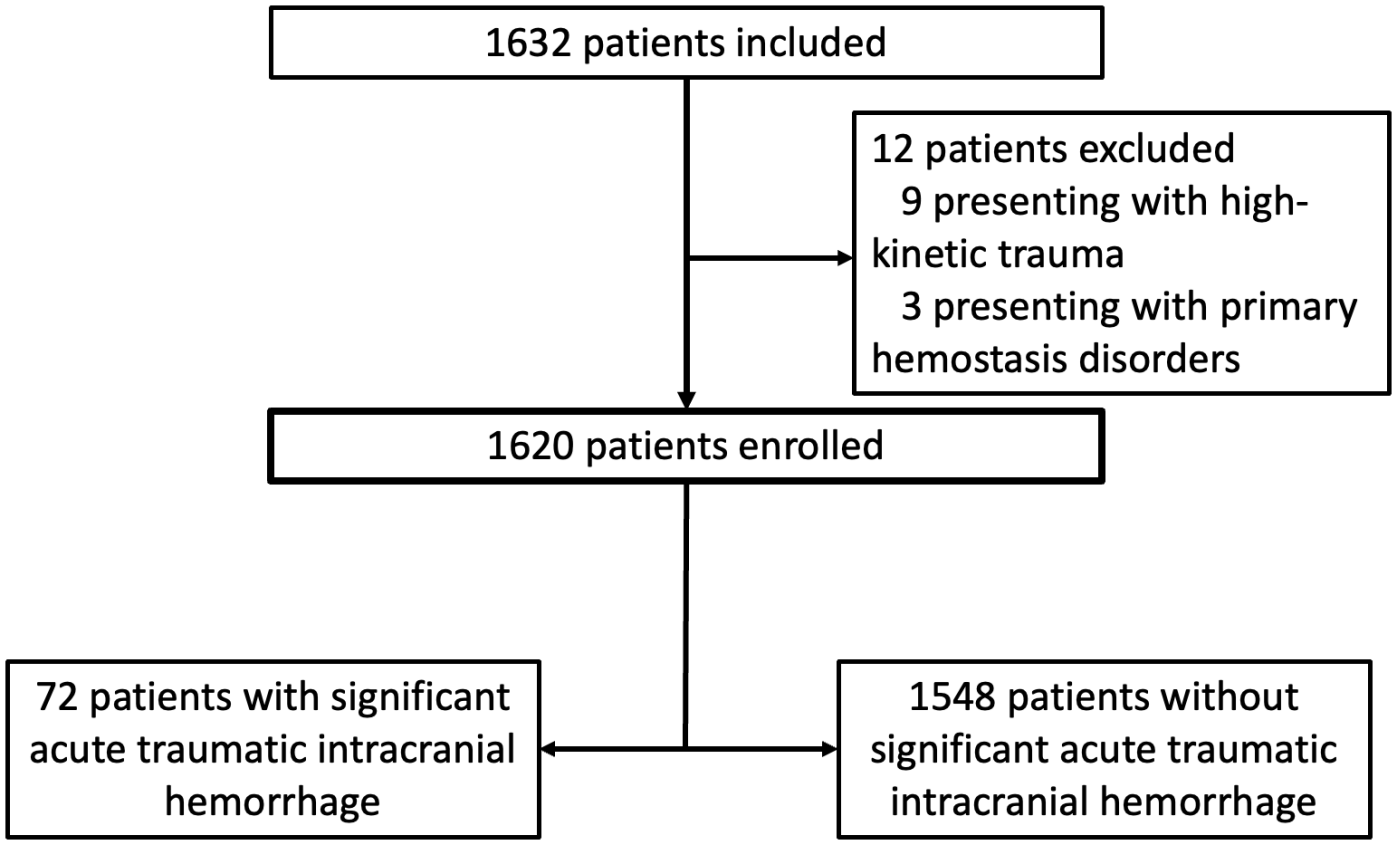
Flow chart.

This study included 1,632 patients examined between 1 July 2023 and 30 June 2025 (inclusion periods for each co-investigating center are provided in S1 Table), of which 12 were excluded (9 due to high-kinetic trauma; 3 due to preinjury primary hemostasis disorders), resulting in a final cohort of 1,620 patients (Fig 1).

The mean age was 84.6 (±8.5) years, 59.9% of the patients were female, most (74.8%) lived at home and 866 (54.4%) were frail (Clinical Frailty Scale score displayed in S2 Table). The median delay between the fall and emergency department presentation was 120 min (IQR: 77–218), and the median delay between emergency department presentation and head CT scan was 157 min (IQR: 94–243). Preinjury antithrombotic medication was reported in 543 (34.8%) patients on antiplatelet agents and 731 (45.1%) on anticoagulants (detailed antithrombotic medication available in S3 Table). Accidental falls were the most common precipitating factor, identified in 911 (56.2%) patients. Four hundred and two (24.8%) patients presented a trauma associated with mild traumatic brain injury (associated trauma details are provided in S4 Table). Additional baseline patient characteristics are presented in Table 1.

**Table 1:** Patient baseline characteristics.

	Overall *N* = 1,620	N (%) Mean (±SD)
Age, years	1,620	84.6 (±8.5)
Sex	1,620	
Male		660 (40.7)
Female		960 (59.3)
Place of residence	1,596	
At home		1,194 (74.8)
Nursing Home		395 (24.8)
Homeless		7 (0.4)
Cognitive impairment	1,620	412 (25.4)
Frailty assessment	1,590	
Robust (CFS 1–3)		545 (34.3)
Frail (CFS 4–6)		866 (54.4)
Severely frail (CFS 7–9)		179 (11.3)
Antiplatelet medication	1,620	563 (34.8)
Single antiplatelet		540 (33.3)
Dual antiplatelet		23 (1.4)
Anticoagulant medication*	1,620	731 (45.1)
Apixaban		450 (61.5)
Rivaroxaban		162 (22.3)
Vitamin K antagonist		59 (8.1)
Parenteral anticoagulant		32 (4.4)
Dabigatran		24 (3.3)
Precipitating factor for falls	1,620	
Accidental		911 (56.2)
Not assessable		405 (25)
Dizziness		151 (9.3)
Syncope		64 (3.9)
Alcohol intoxication		51 (3.2)
Other		38 (2.4)
Post-trauma symptoms	1,620	
None		872 (53.8)
Acute confusion		209 (12.9)
Fall-related amnesia		180 (11.1)
Headache		172 (10.6)
Lost of consciousness		136 (8.4)
Vomiting ≥ 1		83 (5.1)
Not assessable		73 (4.5)
Seizure		15 (0.1)
Physical examination^‡^	1,620	
Glasgow Coma Scale score below baseline		159 (9.8)
Signs of basal skull fracture		61 (3.8)
Pupil abnormalities		47 (2.9)
Focal neurological deficit		27 (1.7)
Visible head impact location	1,620	
Scalp impact		542 (33.5)
Forehead impact		513 (31.6)
Facial impact		178 (11.0)
No visible impact		387 (23.9)
Visible head impact characteristics	1,620	
Bruise		407 (25.1)
Subcutaneous hematoma		472 (29.1)
Wound requiring suture		544 (33.6)

* Four patients with missing data regarding the type of anticoagulant.

‡ Detailed abnormal physical examination available on S5 Table.

Abbreviations: CFS, Clinical Frailty Scale; NA, Not Applicable; SD, Standard Deviation.

Significant acute traumatic intracranial hemorrhage was identified in 72 patients, corresponding to a prevalence of 4.4% (95% CI [3,5]). Among these patients, five (6.9%, 95% CI [2,15]) required neurosurgical intervention, with a median delay of 2 days (interquartile range: 1–4 days). Ten patients (13.9%) had a traumatic brain injury-related death. Subdural hematomas were the most frequently observed lesion, present in 84.7% (*n* = 61/72) of cases, followed by subarachnoid hemorrhage in 43.1% (*n* = 31/72), parenchymal hematoma in 20.1% (*n* = 15/72), and epidural hematoma in 5.6% (*n* = 4/72). Associated with these intracranial hemorrhage, basal skull fractures were found in 5.6% (*n* = 4/72) of patients, and linear vault skull fractures were observed in 11.1% (*n* = 8/72). In the overall cohort, 588 patients (36.3%) were hospitalized. Among those with significant acute traumatic intracranial hemorrhage, 49 patients (68.1%) required hospitalization, while the remaining patients underwent observation in the ED.

In bivariate analysis, compared to patients without visible head impact, patients with facial or forehead impact showed an increased risk of significant acute traumatic intracranial hemorrhage, with an odds ratio of 1.2 (95% CI [0,2]), while those with scalp impact had an odds ratio of 2.8 (95% CI [1,5]). Neither antiplatelet nor anticoagulant use was associated with an increased risk of significant acute traumatic intracranial hemorrhage (Table 2).

**Table 2:** Interobserver agreement and univariate analysis for significant acute traumatic hemorrhage.

	No significant acute traumatic intracranial hemorrhage (*N* = 1,548)	Significant acute traumatic intracranial hemorrhage (*N* = 72)	*p*-value*	Kappa (*N* = 69)
Age, years	84.6 (±8.5)	83.8 (±8.4)	0.45	NA
Sex			0.02	NA
Male	621 (40.1)	39 (54.2)		
Female	927 (59.9)	33 (45.8)		
Cognitive impairment	387 (25.0)	25 (34.7)	0.07	NA
Frailty assessment			0.07	
Robust (CFS 1–3)	528 (34.6)	17 (25.8)		
Frail (CFS 4–6)	821 (53.9)	45 (68.1)		
Severely frail (CFS 7–9)	175 (11.5)	4 (6.1)		
Antiplatelet medication	539 (34.8)	24 (33.3)	0.8	NA
Anticoagulant medication	702 (45.3)	29 (40.3)	0.45	NA
Precipitating factor for falls			0.13	
Environmental	880 (56.8)	31 (43.1)		0.88
Not assessable	381 (24.6)	24 (33.3)		0.76
Dizziness	145 (9.4)	6 (8.3)		0.63
Syncope	60 (3.9)	4 (5.6)		0.79
Alcohol intoxication	46 (3.0)	5 (6.9)		1.0
Other	36 (2.3)	2 (2.8)		0.68
Post trauma symptoms				
None	857 (55.4)	15 (20.1)	<0.001	0.72
Acute confusion	185 (12.0)	24 (33.3)	<0.001	0.67
Fall-related amnesia	170 (11.0)	10 (13.9)	0.44	0.41
Headache	160 (10.3)	23 (32.0)	0.09	0.85
Lost of consciousness	118 (7.6)	18 (25.0)	<0.001	0.63
Vomiting ≥ 1	68 (4.4)	15 (20.8)	<0.001	0.73
Not assessable	72 (4.6)	1 (1.4)	0.2	0.36
Seizure	14 (0.1)	1 (1.4)	0.67	0
Physical examination				
Glasgow Coma Scale score below baseline	134 (8.7)	25 (34.7)	<0.001	0.63
Signs of basal skull fracture	56 (3.6)	5 (6.9)	0.14	0.67
Pupil abnormalities	43 (2.8)	4 (5.6)	0.17	0.71
Focal neurological deficit	20 (1.3)	7 (9.7)	<0.001	0.68
Visible head impact location			<0.001	0.88
Scalp impact	501 (32.4)	41 (56.9)		
Forehead impact	499 (32.2)	14 (19.4)		
Facial impact	172 (11.1)	6 (8.4)		
No visible impact	376 (24.3)	11 (15.3)		
Visible head impact characteristics				
Bruise	385 (24.9)	22 (30.6)	0.27	0.81
Subcutaneous hematoma	446 (28.8)	26 (36.1)	0.18	0.76
Wound requiring suture	524 (33.9)	20 (27.8)	0.28	0.93

*Differences between groups were assessed using Chi-squared or Fisher’s exact test for nominal variables and Mann–Whitney or Wilcoxon *t* test for continuous variables.

Abbreviations: CFS, Clinical Frailty Scale; NA, Not Applicable.

An interobserver agreement calculation was realizable in 69 patients (4.3%). With the exception of amnesia, all other variables (including precipitating factors, post-trauma symptoms, and physical examination findings including visible head impact characteristics and location) showed good reliability, with a kappa coefficient exceeding 0.6.

Logistic regression identified eight predictors that demonstrated good overall accuracy in discriminating cases of significant acute traumatic intracranial hemorrhage, with an area under the receiver operating characteristic curve of 0.91 (Table 3). The models for each step of the logistic regression are available in S6A and S6B Table.

**Table 3:** Final regression model to predict significant acute traumatic intracranial hemorrhage.

	Unadjusted Odd Ratio	95 CI%	Adjusted Odd Ratio	95 CI%
Glasgow Coma Scale score below baseline	5.6	3,9	2.8	1,5
Focal neurological deficit	8.2	3,19	10.5	4,28
Sign of basal skull fracture	2.0	0,5	1.7	0,5
Scalp impact	2.8	1,5	2.6	2,4
Vomiting ≥ 1	5.7	3,10	4.6	2,9
Acute confusion	3.7	2,6	2.6	1,5
Loss of consciousness	4.0	2,7	3.7	2,7
Headache	4.0	2,6	1.8	1,4

Abbreviations: 95% CI, 95% Confidence Interval.

**Fig 2:**
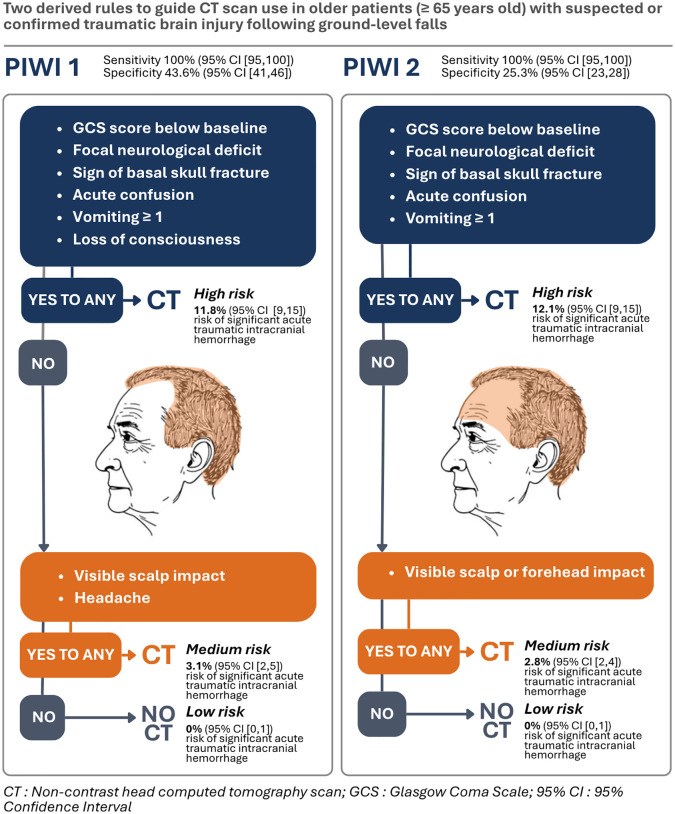
Two derived rules to guide CT scan use in older patients (≥65 years old) with suspected or confirmed traumatic brain injury following ground-level falls.

Subsequent recursive partitioning analysis allowed us to propose two clinical decision rules, both with 100% sensitivity (95% CI [95,100]) (detailed recursive partitioning analysis showed in S1A and S1B Fig). The first one, PIWI 1 rule (Predicting acute traumatic Intracranial hemorrhage with visible head Impact), was devised by incorporating eight predictors derived from fall history and physical examination, and achieved a specificity of 43.6% (95% CI [41,46]). The second rule (PIWI 2) had a lower specificity (25.3%, 95% CI [23,28]) but required only six predictors, all derived from physical examination alone (Fig 2).

Both rules proved reliable, with overall kappa coefficients of, respectively, 0.87 (95% CI [0.7,0.9]) and 0.79 (95% CI [0.7,0.9]). Apparent discrimination performance was good, with C-statistics of, respectively, 0.85 (95% CI [0.8,0.9]) and 0.79 (95% CI [0.7,0.8]). The detailed performance metrics for PIWI 1 and PIWI 2 are presented in Table 4. According to the PIWI 1 rule, 492 patients (30.4%) were in the high-risk group, 453 (28.0%) in the medium-risk group, and 675 (41.7%) in the low-risk group. Under the PIWI 2 rule, 404 patients (24.9%) were classified as high-risk, 824 (50.1%) as medium-risk, and 392 (24.2%) as low-risk. Application of these rules would have reduced CT scan use by, respectively, 41.7% and 24.2%.

**Table 4:** Classification performance.

	Significant acute traumatic intracranial hemorrhage		Sensitivity [95% CI]	Specificity [95% CI]	Negative Predictive Value [95% CI]	Positive Predictive Value [95% CI]	Investigation rate
	Yes	No					
**PIWI 1**			**100%** [95,100]	**43.6%** [41,46]	**100%** [99,100]	**7.6%** [6,9]	**58.3%**
Positive	72	873					
Negative	0	675					
**PIWI 2**			**100%** [95,100]	**25.3%** [23,28]	**100%** [99,100]	**5.9%** [5,7]	**75.8%**
Positive	72	1,156					
Negative	0	392					

Abbreviation: 95% CI, 95% Confidence Interval.

Internal validation confirmed the good performance of both rules. For PIWI 1 rule, the bootstrap procedure estimated a C-statistic of 0.84 (95% CI [0.8,0.9]), indicating good discrimination. Calibration was adequate, with a calibration-in-the-large of −0.005 (95% CI [−0.3,0.3]) and a calibration slope of 0.91 (95% CI [0.8,1]). For PIWI 2 rule, the C-statistic was 0.79 (95% CI [0.7,0.8]), with a calibration-in-the-large of 0.005 (95% CI [−0.2,0.3]) and a calibration slope of 0.92 (95% CI [0.7,1]). The calibration plots for both models, showing the agreement between observed and expected risks of intracranial lesions across deciles of predicted risk, are available in S2A and S2B Fig.

## Discussion

In this prospective cohort study involving 1,620 older patients presenting with symptoms of mild traumatic brain injury due to ground-level falls, we identified a set of criteria that indicate likely significant acute traumatic intracranial hemorrhage and thus justify ordering a head CT scan. According to our findings, scans could be performed selectively in patients exhibiting one or more of the following eight factors: a Glasgow Coma Scale below baseline, focal neurological deficit, signs of basal skull fracture, visible scalp or forehead impact, vomiting, acute confusion, loss of consciousness, or headache. Implementation of decision rules derived therefrom, namely PIWI 1 or PIWI 2, could reduce referrals to the CT scan unit by, respectively, 41.7% or 24.2%.

Consistent with previous studies, our study population included primarily frail older adults, with one in four patients exhibiting preinjury cognitive impairment. Given the frequent unreliability or unavailability of fall history details, such as precipitating factors and post-trauma symptoms, we developed two clinical decision rules to compensate for the absence of such information. The first rule, PIWI 1, was designed to maximize specificity but includes variables related to fall history, such as loss of consciousness, which can be difficult to assess consistently in this population. The second rule, PIWI 2, less specific, is based exclusively on objective clinical findings and excludes subjective symptoms like headache, pain and related symptoms that are difficult to evaluate in patients with cognitive impairment or unclear histories. Our goal was to develop rules applicable specifically to our population of interest while offering a pragmatic approach to physicians for routine practice.

Regarding the clinical findings included in these rules, this study confirmed our hypothesis on the role of visible head impact location in assessing the likelihood that traumatic hemorrhage has occurred [18–20]. Such use of this information had only been suggested in a single retrospective study [37]. Similar results have been reported in pediatric populations. In a cohort of children aged 0–16 years presenting to the emergency department with mild traumatic brain injury, temporal-parietal and occipital cutaneous impact locations were associated with an increased likelihood of traumatic hemorrhage [38]. In adult patients with mild traumatic brain injury, the presence of a scalp hematoma has been found associated with an increased likelihood of traumatic intracranial hemorrhage and is therefore included in the NEXUS decision rule [39]. This association suggests several hypotheses. One is that the lack of adaptive postural responses to falls in older patients (frail in particular) may play a role: despite the low kinetic energy of the impact, the absence of protective upper limb reflexes could lead directly to brain injury and hence an increased likelihood of traumatic hemorrhage. One study has focused on specifics of postural reflexes of older fall victims [40]. Another avenue to examine is specific anatomical features of the aging brain that may increase likelihood. The formation of a subdural hematoma (the leading cause of traumatic hemorrhage in this population) involves direct trauma capable of damaging the venous network of the dura mater and arachnoid [41]. In older patients, increased vulnerability of vascular tissue (linked to structural changes in white matter) may explain the heightened susceptibility to hemorrhagic lesions, even in low-kinetic-energy trauma. Biomechanical studies may provide better understanding of the underlying mechanisms in this population [42]. Glasgow Coma Scale score below baseline, focal neurological deficit, and signs of basal skull fracture have been associated previously with high likelihood of significant acute traumatic intracranial hemorrhage [10,11,39]. The inclusion of headache in the rule may be debatable. In this population, it can indeed be challenging to distinguish between diffuse headache and focal headache related to visible head impact. However, this variable displayed excellent interobserver agreement (*κ* = 0.85), which most physicians would find compelling. The same consideration applies to loss of consciousness. In our preliminary study, both loss of consciousness and fall-related amnesia were associated with an increased likelihood of traumatic hemorrhage [18]. However, in clinical practice, it may be difficult to differentiate syncope from loss of consciousness and fall-related amnesia in some patients. This is why these historical factors were not included in the second rule (PIWI 2). Although it may seem counterintuitive, antithrombotic medication was not associated with an increased risk of traumatic hemorrhage in our study population. This result is consistent with recent studies focused such patients [16].

Over the past decade, emergency department visits for fall-related traumatic brain injury among older patients have more than doubled in the USA, with similar trends observed in European countries [9,43]. In addition to reducing radiation exposure and healthcare costs, a more targeted strategy for CT scanning is needed to improve patient care pathways. In some settings, 50% of the waiting time in the emergency department may be spent in the queue for head CT scans [44]. The present study provides a novel approach to assessing older patients with mild traumatic brain injury sustained by a ground-level fall. Based on simple and reliable criteria, a large subgroup of these patients would not require a head CT scan to exclude traumatic intracranial hemorrhage. External validation of this rule is now necessary before it can be implemented in clinical practice, but these findings may already help identify high-risk patients. We hypothesize that the PIWI 1 rule could be used in patients with a clear history of the fall, while the PIWI 2 rule could be applied in other cases; this hypothesis should be tested during the validation stage. Moreover, based on this risk stratification, further studies could explore the use of biomarkers as an alternative to CT scans in the high- and medium-risk groups.

Among the limitations of this study, the risk of selection bias must be considered, since we included only patients who underwent a head CT scan during their emergency department stay. Patients who were not scanned (and may have had undetected traumatic hemorrhage) were excluded. However, we assume that this risk is minimal, since our preliminary study indicated that most (75.2%) of these patients were scanned [28]. In addition, all emergency physicians adhered to national and European guidelines for mild traumatic brain injury, which recommend broad usage of CT scans in this population, suggesting that unscreened patients were unlikely to have suffered traumatic lesions [45]. There is also a risk of misclassification bias, because we did not perform centralized re-reading of the scans. However, all scans were interpreted by a neuroradiologist, and previous studies have shown that interpretations in usual care settings are reliable [46]. Third, the choice of the NIRIS score to define the primary outcome may be debatable [32]. It has not been validated in older populations, but at the time was the only validated scoring system for traumatic brain injury [30,31]. Fourth, there is a potential risk of diagnostic bias. We may not have detected the risk of delayed bleeding after the initial CT scan, which though minimal does exist in this population [33]. Similarly, neurosurgical intervention may have occurred later than our day 7 follow-up. However, in this population, such procedures almost always take place within a few days after injury [47]. Regarding the statistical analysis, 72 patients had significant acute traumatic intracranial hemorrhage, compared to the 80 patients estimated during the sample size calculation. This lower-than-expected prevalence may increase the risk of overfitting in the multivariate models.

Our findings revealed that factors drawn from patient history and physical examination, integrating visible head impact location, were associated with significant acute traumatic intracranial hemorrhage in older adults after a ground-level fall. Incorporating these factors into decision rules could provide a reliable strategy to stratify risk and reduce unnecessary CT scan, thereby limiting healthcare costs and needless radiation exposure. Such rules need to be validated externally and independently before being proposed for implementation in clinical practice. Nevertheless, these rules may already be helpful in identifying high-risk patients.

### Transparency

The lead author (XD) affirms that the manuscript is an honest, accurate, and transparent account of the study being reported and that no important aspects of the study have been omitted. Both XD and MÉ had full access to all the data in the study. The corresponding author had final responsibility for the decision to submit the manuscript for publication.

### Dissemination to participants and related patient and public communities

Results of the study will be shared with policy makers, professional societies, and the public through press release and social media.

## Supporting information

S1 Checklist STROBE. Adapted from the STROBE Statement, available under a Creative Commons Attribution 4.0 International License (CC BY 4.0) at https://www.strobe-statement.org/ DOI: https://doi.org/10.1016/j.jclinepi.2007.11.008.
(DOCX)

S2 Checklist TRIPOD. Adapted from the TRIPOD Statement, available under a Creative Commons Attribution 4.0 International License (CC BY 4.0) at https://www.tripod-statement.org/ DOI: https://doi.org/10.7326/M14-0697.
(DOCX)

S1 Table Inclusion periods for each co-investigating center and number of inclusions. (DOCX)

S2 Table Clinical Frailty Scale assessment. (DOCX)

S3 Table Antithrombotic medication. (DOCX)

S4 Table Detailed trauma associated with traumatic brain injury. (DOCX)

S5 Table Detailed abnormal physical examination. (DOCX)

S6 Table Table A. Complete model logistic regression. **Table B.** Final model logistic regression.
(DOCX)

S1 Fig Fig A. Recursive partitioning. PIWI 1 rule. **Fig B.** Recursive partitioning. PIWI 2 rule.
(DOCX)

S2 Fig Fig A. Calibration plot. PIWI 1. **Fig B.** Calibration plot. PIWI 2.
(DOCX)

S1 File Study protocol. (DOCX)

## Abbreviations

AIC: Akaike Information Criterion
CT: computed tomography
CTHEAD: Computed Tomography of the Head for Patients at Advanced Age
GDPR: General Data Protection Regulation
NIRIS: Neuroimaging Radiological Interpretation System
STROBE: Strengthening the Reporting of Observational Studies in Epidemiology
TRIPOD: Transparent Reporting of a Multivariable Prediction Model for Individual Prognosis Or Diagnosis
