# Supplementary material for: Risk stratification for significant acute traumatic intracranial hemorrhage in older adults after a ground-level fall: A prospective multicentre cohort study
Source: PLoS Med. 2026 Aug 10;23(8):e1004935. doi: 10.1371/journal.pmed.1004935 (PMC13456197; doi:10.1371/journal.pmed.1004935)
Supplement: Supplementary file 1 [file pmed.1004935.s001.docx]

**S1 Checklist – STROBE***

|  | Item No | Recommendation |  |
| --- | --- | --- | --- |
| **Title and abstract** | 1 | (*a*) Indicate the study’s design with a commonly used term in the title or the abstract | Title |
|  |  | (*b*) Provide in the abstract an informative and balanced summary of what was done and what was found | Abstract |
| Introduction | | |  |
| Background/rationale | 2 | Explain the scientific background and rationale for the investigation being reported | Introduction, first paragraph |
| Objectives | 3 | State specific objectives, including any prespecified hypotheses | Introduction, third paragraph |
| Methods | | |  |
| Study design | 4 | Present key elements of study design early in the paper | Methods, study design |
| Setting | 5 | Describe the setting, locations, and relevant dates, including periods of recruitment, exposure, follow-up, and data collection | Methods, study design & participants |
| Participants | 6 | (*a*) *Cohort study*—Give the eligibility criteria, and the sources and methods of selection of participants. Describe methods of follow-up  *Case-control study*—Give the eligibility criteria, and the sources and methods of case ascertainment and control selection. Give the rationale for the choice of cases and controls  *Cross-sectional study*—Give the eligibility criteria, and the sources and methods of selection of participants | Methods, study design & participants |
|  |  | (*b*) *Cohort study*—For matched studies, give matching criteria and number of exposed and unexposed  *Case-control study*—For matched studies, give matching criteria and the number of controls per case |  |
| Variables | 7 | Clearly define all outcomes, exposures, predictors, potential confounders, and effect modifiers. Give diagnostic criteria, if applicable | Methods, Data collection |
| Data sources/ measurement | 8* | For each variable of interest, give sources of data and details of methods of assessment (measurement). Describe comparability of assessment methods if there is more than one group | Methods, Data collection |
| Bias | 9 | Describe any efforts to address potential sources of bias | Methods, Data collection |
| Study size | 10 | Explain how the study size was arrived at | Methods, Sample size |
| Quantitative variables | 11 | Explain how quantitative variables were handled in the analyses. If applicable, describe which groupings were chosen and why | Methods, Statistical analysis |
| Statistical methods | 12 | (*a*) Describe all statistical methods, including those used to control for confounding | Methods, Statistical analysis |
|  |  | (*b*) Describe any methods used to examine subgroups and interactions | Methods, Statistical analysis |
|  |  | (*c*) Explain how missing data were addressed | Methods, Statistical analysis |
|  |  | (*d*) *Cohort study*—If applicable, explain how loss to follow-up was addressed  *Case-control study*—If applicable, explain how matching of cases and controls was addressed  *Cross-sectional study*—If applicable, describe analytical methods taking account of sampling strategy | Methods, Statistical analysis |
|  |  | (*e*) Describe any sensitivity analyses |  |

Continued on next page

| Results | | |  |
| --- | --- | --- | --- |
| Participants | 13* | (a) Report numbers of individuals at each stage of study—eg numbers potentially eligible, examined for eligibility, confirmed eligible, included in the study, completing follow-up, and analysed | Results |
|  |  | (b) Give reasons for non-participation at each stage | Results – flow-chart |
|  |  | Results | Results – flow chart |
| Descriptive data | 14* | (a) Give characteristics of study participants (eg demographic, clinical, social) and information on exposures and potential confounders | Results – first paragraph |
|  |  | (b) Indicate number of participants with missing data for each variable of interest | Resultats – table 1 |
|  |  | (c) *Cohort study*—Summarise follow-up time (eg, average and total amount) | Results – first paragraph |
| Outcome data | 15* | *Cohort study*—Report numbers of outcome events or summary measures over time | Results – second paragraph |
|  |  | *Case-control study—*Report numbers in each exposure category, or summary measures of exposure |  |
|  |  | *Cross-sectional study—*Report numbers of outcome events or summary measures |  |
| Main results | 16 | Give unadjusted estimates and, if applicable, confounder-adjusted estimates and their precision (eg, 95% confidence interval). Make clear which confounders were adjusted for and why they were included | Results – third paragraph |
|  |  | (*b*) Report category boundaries when continuous variables were categorized | N/A |
|  |  | (*c*) If relevant, consider translating estimates of relative risk into absolute risk for a meaningful time period | Results – third paragraph |
| Other analyses | 17 | Report other analyses done—eg analyses of subgroups and interactions, and sensitivity analyses | Results – third paragraph |
| Discussion | | |  |
| Key results | 18 | Summarise key results with reference to study objectives | Discussion – first paragraph |
| Limitations | 19 | Discuss limitations of the study, taking into account sources of potential bias or imprecision. Discuss both direction and magnitude of any potential bias | Discussion – second paragraph |
| Interpretation | 20 | Give a cautious overall interpretation of results considering objectives, limitations, multiplicity of analyses, results from similar studies, and other relevant evidence | Discussion – limitation |
| Generalisability | 21 | Discuss the generalisability (external validity) of the study results | Discussion - conclusion |
| Other information | | |  |
| Funding | 22 | Give the source of funding and the role of the funders for the present study and, if applicable, for the original study on which the present article is based | Funding |

*** STROBE Statement.** Checklist of items that should be included in reports of cohort studies. *Adapted from the STROBE Statement, available under a Creative Commons Attribution 4.0 International License (CC BY 4.0) at* [*https://www.strobe-statement.org*](https://www.strobe-statement.org) */ DOI:* [*https://doi.org/10.1016/j.jclinepi.2007.11.008*](https://www.google.com/search?q=https://doi.org/10.1016/j.jclinepi.2007.11.008)
