# Supplementary material for: Risk stratification for significant acute traumatic intracranial hemorrhage in older adults after a ground-level fall: A prospective multicentre cohort study
Source: PLoS Med. 2026 Aug 10;23(8):e1004935. doi: 10.1371/journal.pmed.1004935 (PMC13456197; doi:10.1371/journal.pmed.1004935)
Supplement: Supplementary file 2 [file pmed.1004935.s002.docx]

**S2 Checklist – TRIPOD***

***TRIPOD Statement.** Transparent Reporting of a multivariable prediction model for Individual Prognosis Or Diagnosis. *Adapted from the TRIPOD Statement, available under a Creative Commons Attribution 4.0 International License (CC BY 4.0) at* [*https://www.tripod-statement.org*](https://www.tripod-statement.org) */ DOI:* [*https://doi.org/10.7326/M14-0697*](https://www.google.com/search?q=https://doi.org/10.7326/M14-0697)
