# Supplementary material for: Risk stratification for significant acute traumatic intracranial hemorrhage in older adults after a ground-level fall: A prospective multicentre cohort study
Source: PLoS Med. 2026 Aug 10;23(8):e1004935. doi: 10.1371/journal.pmed.1004935 (PMC13456197; doi:10.1371/journal.pmed.1004935)
Supplement: Supplementary file 3 [file pmed.1004935.s003.docx]

**S1 Table. Inclusion periods for each co-investigating center and number of inclusions**

| **Center** | **Annual Emergency Department Volume in 2024** | **Inclusion period** | **Number of inclusions** |
| --- | --- | --- | --- |
| **Toulouse University Hospital Center, Toulouse, France** | 138,000 visits | **From July 1, 2023, to June 30, 2025** | 620 patients |
| **Poitiers University Hospital Center, Poitiers, France** | 50,000 visits | **From August 1, 2023, to June 30, 2025** | 455 patients |
| **Cochin University Hospital Center, Paris, France** | 65,000 visits | **From November 1, 2023, to June 30, 2025** | 386 patients |
| **Louis Pasteur University Hospital, Kosice, Slovakia** | 34,000 visits | **From April 1, 2024, to June 30, 202** | 130 patients |
| **Princesse Grace Hospital Center, Avenue Pasteur, Monte Carlo, Monaco.** | 65,000 visits | **From July 1, 2024, to June 30, 2025** | 41 patients |
| **TOTAL** | 352,000 visits | **From July 1, 2023, to June 30, 2025** | 1632 patients |
