# Supplementary material for: Risk stratification for significant acute traumatic intracranial hemorrhage in older adults after a ground-level fall: A prospective multicentre cohort study
Source: PLoS Med. 2026 Aug 10;23(8):e1004935. doi: 10.1371/journal.pmed.1004935 (PMC13456197; doi:10.1371/journal.pmed.1004935)
Supplement: Supplementary file 4 [file pmed.1004935.s004.docx]

**S2 Table. Clinical Frailty Scale assessment**

|  |  | 1590* |
| --- | --- | --- |
| 1 - Very fit |  | 83 (5.2) |
| 2 - Fit |  | 190 (11.9) |
| 3 - Managing well |  | 272 (17.1) |
| 4 - Living with very mild frailty |  | 339 (21.3) |
| 5 - Living with mild frailty |  | 284 (17.9) |
| 6 - Living with moderate frailty |  | 243 (15.3) |
| 7 - Living with severe frailty |  | 166 (10.4) |
| 8 - Living with very severe frailty |  | 12 (0.8) |
| 9 – Terminally ill |  | 1 (0.1) |

*30 patients with missing data
