# Supplementary material for: Risk stratification for significant acute traumatic intracranial hemorrhage in older adults after a ground-level fall: A prospective multicentre cohort study
Source: PLoS Med. 2026 Aug 10;23(8):e1004935. doi: 10.1371/journal.pmed.1004935 (PMC13456197; doi:10.1371/journal.pmed.1004935)
Supplement: Supplementary file 5 [file pmed.1004935.s005.docx]

**S3 Table. Antithrombotic medication**

| Antiplatelet |  | 563 |
| --- | --- | --- |
| Acetylsalicylic acid |  | 489 (86.7) |
| Clopidogrel |  | 90 (15.9) |
| Ticagrelor |  | 4 (0.7) |
| Anticoagulant* |  | 731 |
| Apixaban |  | 450 (61.5) |
| Rivaroxaban |  | 162 (22.3) |
| Vitamine K antagonist |  | 59 (8.1) |
| Parentéral anticoagulant |  | 32 (4.4) |
| Dabigatran |  | 24 (3.3) |

**4 patients with unknown anticoagulant medication*
