# Supplementary material for: Risk stratification for significant acute traumatic intracranial hemorrhage in older adults after a ground-level fall: A prospective multicentre cohort study
Source: PLoS Med. 2026 Aug 10;23(8):e1004935. doi: 10.1371/journal.pmed.1004935 (PMC13456197; doi:10.1371/journal.pmed.1004935)
Supplement: Supplementary file 6 [file pmed.1004935.s006.docx]

**S4 Table. Detailed trauma associated with traumatic brain injury**

| Trauma associated with traumatic brain injury |  | 402 |
| --- | --- | --- |
| Bruise or laceration other than head injury |  | 128 (31.8) |
| Facial fracture |  | 99 (24.6) |
| Upper limb fracture |  | 81 (20.1) |
| Lower limb fracture |  | 62 (15.4) |
| Thoracolumbar spine or pelvic fracture |  | 23 (5.7) |
| Cervical spine fracture |  | 16 (4) |
| Rib fracture(s) |  | (3.5) |
| Other fracture |  | 26 (6.5) |
