# Supplementary material for: Risk stratification for significant acute traumatic intracranial hemorrhage in older adults after a ground-level fall: A prospective multicentre cohort study
Source: PLoS Med. 2026 Aug 10;23(8):e1004935. doi: 10.1371/journal.pmed.1004935 (PMC13456197; doi:10.1371/journal.pmed.1004935)
Supplement: Supplementary file 7 [file pmed.1004935.s007.docx]

**S5 Table. Detailed abnormal physical examination**

| Reduced Glasgow Coma scale from baseline | |  | 159 |
| --- | --- | --- | --- |
| 14 |  | | 97 |
| 13 |  | | 24 |
| *Missing data* |  | | 38 |
| Signs of basal skull fracture |  | | **61** |
| Periorbital echymosis |  | | 47 |
| Otorrhagia |  | | 8 |
| Otoorrhea |  | | 1 |
| *Missing data* |  | | 5 |
| Focal neurological sign |  | | 27 |
| Focal motor deficit |  | | 14 |
| Aphasia |  | | 6 |
| Cerebellar syndrome |  | | 1 |
| *Missing data* |  | | 6 |
| Pupil abnormalities |  | | 27 |
| Anisocoria |  | | 20 |
| Miosis |  | | 7 |
