# Supplementary material for: Risk stratification for significant acute traumatic intracranial hemorrhage in older adults after a ground-level fall: A prospective multicentre cohort study
Source: PLoS Med. 2026 Aug 10;23(8):e1004935. doi: 10.1371/journal.pmed.1004935 (PMC13456197; doi:10.1371/journal.pmed.1004935)
Supplement: Supplementary file 8 [file pmed.1004935.s008.docx]

**S6A Table. Complete model logistic regression**

Abbrevations: homme, sex mal; fp_meca, environmental fall; fp_intoxicationOH, alcohol intoxication; fp_syncope, syncope; sf_amnesie, Fall-related amnesia; sf_cephalee, headache; sf_confusion, acute confusion; sf_pc, loss of consciousness; sf_vomissement, vomiting; alteration_vigilance, Glasgow Coma Score below baseline; deficit_neurologique, focal neurological deficit; pupille, pupil abnormality; suspi_frbasecrane, sign of basal skull fracture; locimpact, visible head impact location; hematoma, subcutaneous hemoatoma; plaiesuturer: wound requiring stiches.

**S6B Table. Final model logistic regression**

Abbrevations: sf_cephalee, headache; sf_confusion, acute confusion; sf_pc, loss of consciousness; sf_vomissement, vomiting; alteration_vigilance, Glasgow Coma Score below baseline; deficit_neurologique, focal neurological deficit; suspi_frbasecrane, sign of basal skull fracture; locimpact, visible head impact location; hematoma
