# Supplementary material for: Risk stratification for significant acute traumatic intracranial hemorrhage in older adults after a ground-level fall: A prospective multicentre cohort study
Source: PLoS Med. 2026 Aug 10;23(8):e1004935. doi: 10.1371/journal.pmed.1004935 (PMC13456197; doi:10.1371/journal.pmed.1004935)
Supplement: Supplementary file 9 [file pmed.1004935.s009.docx]

**S1A Figure. Recursive partitioning. PIWI 1 rule**

Abbrevations: GCS, Glasgow Coma Scale score

**S1B Figure. Recursive partitioning. PIWI 2 rule**

Abbrevations: GCS, Glasgow Coma Scale score
