# Supplementary material for: Risk stratification for significant acute traumatic intracranial hemorrhage in older adults after a ground-level fall: A prospective multicentre cohort study
Source: PLoS Med. 2026 Aug 10;23(8):e1004935. doi: 10.1371/journal.pmed.1004935 (PMC13456197; doi:10.1371/journal.pmed.1004935)
Supplement: Supplementary file 11 [file pmed.1004935.s011.docx]

**S1 File: Study Protocol**

Risk stratification for significant acute traumatic intracranial hemorrhage in older adults after a ground-level fall: A prospective multicentre cohort study

Stratification du risque de lésion hémorragique intracérébrale traumatique aiguë significative chez les patients âgés suite à une chute de leur hauteur : une étude de cohorte prospective multicentrique

**PIWI**

**(Predicting Intracranial hemorrhage With visible head Impact)**

Research protocol not involving human participants and compliant with the MR-004 reference methodology

**Version No. 1 dated 05/10/2023**

Administrator

###### **CHU TOULOUSE**

Hôtel Dieu

2, rue Viguerie TSA 80035

31059 Toulouse cedex 9

Investigator

###### **Dr Xavier Dubucs**

Emergency Department

CHU Toulouse Purpan

1 place du Dr Baylac

31059 Toulouse Cedex 9

| **Administrator** | CHU Toulouse |
| --- | --- |
| **Investigator** | Dr Xavier Dubucs |
| **Title** | Risk stratification for significant acute traumatic intracranial hemorrhage in older adults after a ground-level fall: A prospective multicentre cohort study |
| **Background** | In industrialized nations over the past thirty years, the median age for the occurrence of traumatic brain injury (TBI) has nearly doubled. However, there is currently no predictive model for traumatic intracranial hemorrhage (TIH) specifically validated for this older population. The application of currently available clinical decision rules leads to the systematic performance of a non-contrast head computed tomography (CT) scan for all older patients admitted to the emergency department for TBI. Indeed, a head CT scan remains the gold standard for visualizing TIH. All patients over 65 years of age who experience a mild TBI (mTBI) must undergo an emergency head CT scan within 4 hours post-injury. This rule is also advocated by the 2012 formalized expert guidelines. Yet, recent literature suggests that the prevalence of TIH in older patients admitted after a ground-level fall is low; only 5.2% of head CT scans performed in this context reveal a TIH, and less than one percent of these patients require neurosurgical intervention. This low prevalence of TIH is partly explained by the low-kinetic mechanism (ground-level fall) of the TBI and by the systematic performance of head CT scans. When contrasting the recent increase in emergency department visits for this reason among older adults with their low representation in the international literature, this paradox is likened to a "silent epidemic." Antiplatelet agents are frequently prescribed in adults and older individuals, with some studies estimating their use by half of the Western population. While antiplatelet intake is a widely accepted risk factor for TIH following an mTBI and constitutes an indication for an emergency head CT scan, the excess risk of TIH associated with antiplatelets has recently been challenged in the general population presenting with mTBI. Probst et al. (2020) demonstrated in a prospective emergency department-based study that antiplatelet monotherapy was not associated with an increased risk of TIH in TBI cases [1]. A meta-analysis published by Fiorelli et al. in 2020 tempered this finding by identifying a slight increase in TIH risk, estimated at 1.51, particularly among older adults [2]. In contrast, the only available prospective multicenter cohort study, conducted by De Wit et al., which focused exclusively on older adults experiencing ground-level falls, did not find this excess risk of TIH [3].  A similar observation applies to anticoagulant therapy (vitamin K antagonists or direct oral anticoagulants): while there is a proven increased risk of TIH in the general population following a TBI, this heightened risk is not observed when focusing solely on older adults who fall from standing height. Corroborating these findings, our study conducted at the Toulouse University Hospital showed no excess risk of TIH in this population associated with the use of either antiplatelets or anticoagulants.  Furthermore, there are likely risk factors for TIH that are specific to this population. For example, Timler et al. observed that the mechanism of the fall, such as syncope, was associated with an increased risk of TIH compared to other mechanisms [4]. Integrating these clinical data and geriatric characteristics is necessary to better identify the risk factors for TIH in this population. In 2023, we conducted a retrospective cohort study validating specific risk factors for this population. This was a single-center study conducted at the Toulouse University Hospital. In the multivariate analysis, the location of the impact, the presence of a subcutaneous hematoma, vomiting, amnesia of the fall, loss of consciousness, and altered mental status were associated with an increased risk of TIH in older patients admitted for head trauma following a ground-level fall.  Our research hypothesis is that these potential risk factors for TIH, identified retrospectively, will be validated prospectively and multicenter. This represents a crucial step prior to the development of a clinical decision rule for predicting TIH risk in this population, in order to better target the performance of a head CT scan. |
| **Objectives** | **Primary objective:** To identify risk factors for significant acute traumatic intracranial hemorrhage following a ground-level fall with head trauma in older patients admitted to the emergency department.  **Secondary objectives:**   - To evaluate the prevalence of emergency neurosurgical intervention. - To characterize the post-emergency department outcomes of these patients. |
| **Study design** | prospective, multicenter, observational cohort study |
| **Inclusion Criteria** | - Presenting to an emergency department with a mild traumatic brain injury (mTBI) due to a ground-level fall (from a standing position, off a chair, or out of bed) occurring up to 24 hours prior to arrival. - Having undergone a head CT scan. - mTBI defined broadly as head trauma with a Glasgow Coma Scale (GCS) score between 13 and 15 upon presentation, presumed present if a head impact was reported (by patient or witness) and/or external signs of head trauma were noted by the clinician. (Transient neurological symptoms like loss of consciousness were not required due to high rates of baseline cognitive impairment). - Patients on antithrombotic therapy (antiplatelet or anticoagulant agents) were eligible. |
| **Exclusion criteria** | - High-kinetic trauma (e.g., collisions involving motorized vehicles or bicycles). - Patients with hemophilia (congenital or acquired). - Patients with primary hemostasis disorders (e.g., von Willebrand disease, immune thrombocytopenic purpura). |
| **Outcomes** | ** Primary outcome:** Significant acute traumatic intracranial hemorrhage based on a head CT scan. It will be defined by a Neuroimaging Radiological Interpretation System (NIRIS) score greater than 1 (including epidural, subdural, or parenchymal hematoma, midline shift, etc.). A single CT scan will be used to rule out hemorrhage, even for patients on antithrombotic medication. Results will be reviewed by blinded local neuroradiologists and recorded in REDCap by a blinded investigator.  ** Secondary outcomes:** Any neurosurgical intervention or in-hospital death related to traumatic brain injury occurring within seven days of the emergency department presentation. |
| **Sample Size** | 1600 patients |
| **Research duration** | **Data collection start:** June 2023  **Data collection duration:** 18 months  **Total study duration:** 24 months |
| **Statistical analysis** | We will develop a clinical decision rule whereby significant acute traumatic intracranial hemorrhage will be presumed excludable in the absence of any risk factors. If one or more risk factors are present, the rule will recommend performing a CT scan. This rule will include the minimal number of predictor variables required to achieve 100% sensitivity. First, bivariate analyses will be conducted to assess the strength of association between each potential predictor and the primary outcome, using Chi-square or Fisher’s exact test for nominal variables and Mann-Whitney or Wilcoxon t-test for continuous variables, as appropriate. Predictors included in the rule will then be identified through logistic regression and recursive partitioning. Different combinations of the visible head impact variable will be tested during these analyses (e.g., scalp impact vs. other; forehead-scalp impact vs. other). A stepwise backward selection process, based on p-values and the Akaike Information Criterion (AIC), will be used to refine the predictive factors in the final regression model. Variables with a p-value < 0.25 in logistic regression will be further evaluated using recursive partitioning analysis. The interobserver agreement for each variable will be assessed using the kappa coefficient or Spearman’s interclass correlation coefficient. A predictor will be included in the rule only if it is both strongly associated with the primary outcome and displays good interobserver reliability (kappa > 0.6). The reliability of each clinical decision rule will be evaluated by measuring the interobserver agreement for all predictors included in the rule. Our goal will be to develop a clinically relevant, reliable, and easily implementable decision rule for routine practice.The prediction model will be assessed first on apparent performance. The C-statistic will be calculated to measure model discrimination ability. Internal validation will be performed using bootstrapping. Five hundred bootstrap samples will be generated to assess performance of the final model. The C-Statistic will be calculated also to estimate the area under the receiver operating characteristics curve of the final model on the bootstrap samples. The calibration of the final model will be assessed by estimating calibration-in-the-large and the calibration slope. The calibration plot will be generated to illustrate the agreement. |

References

1.	Probst MA, Gupta M, Hendey GW, Rodriguez RM, Winkel G, Loo GT, et al. Prevalence of Intracranial Injury in Adult Patients With Blunt Head Trauma With and Without Anticoagulant or Antiplatelet Use. Annals of Emergency Medicine. 2020;75: 354–364. doi:cushi

2.	Fiorelli EM, Bozzano V, Bonzi M, Rossi SV, Colombo G, Radici G, et al. Incremental Risk of Intracranial Hemorrhage After Mild Traumatic Brain Injury in Patients on Antiplatelet Therapy: Systematic Review and Meta-Analysis. J Emerg Med. 2020;59: 843–855. doi:10.1016/j.jemermed.2020.07.036

3.	de Wit K, Parpia S, Varner C, Worster A, McLeod S, Clayton N, et al. Clinical Predictors of Intracranial Bleeding in Older Adults Who Have Fallen: A Cohort Study. Journal of the American Geriatrics Society. 2020;68: 970–976. doi:10.1111/jgs.16338

4.	Timler D, Dworzyński MJ, Szarpak Ł, Gaszyńska E, Dudek K, Gałazkowski R. Head trauma in elderly patients: Mechanisms of injuries and CT findings. Advances in Clinical and Experimental Medicine. 2015;24: 1045–1050. doi:10.17219/acem/27565
