# Supplementary material for: Risk stratification for significant acute traumatic intracranial hemorrhage in older adults after a ground-level fall: A prospective multicentre cohort study
Source: PLoS Med. 2026 Aug 10;23(8):e1004935. doi: 10.1371/journal.pmed.1004935 (PMC13456197; doi:10.1371/journal.pmed.1004935)
Supplement: Supplementary file 12 [file pmed.1004935.s010.docx]

**S2A Figure. Calibration plot. PIWI 1**

**S2B Figure. Calibration plot. PIWI 2**
