# Supplementary material for: Risk stratification for significant acute traumatic intracranial hemorrhage in older adults after a ground-level fall: A prospective multicentre cohort study
Source: PLoS Med. 2026 Aug 10;23(8):e1004935. doi: 10.1371/journal.pmed.1004935 (PMC13456197; doi:10.1371/journal.pmed.1004935)
Supplement: Supplementary file 13 [file pmed.1004935.s014.docx]

April 12th, 2026

To Katarzyna Starowicz, Associate Editor
Plos Medicine

Ref.:  PMEDICINE-D-26-00283R1
Title: Risk stratification for significant acute traumatic intracranial hemorrhage in older adults after a ground-level fall: a prospective multicentre cohort study

Dear Associate Editor,

Thank you for offering us the opportunity to revise our manuscript “Risk stratification for significant acute traumatic intracranial hemorrhage in older adults after a ground-level fall: a prospective multicentre cohort study”. We have revised the paper according to the Reviewer’s comments and Journal Requirements. In this letter, we address the changes made point by point.

On behalf of all authors,

Xavier Dubucs

Journal Requirements:

1) Please ensure that the funders and grant numbers match between the Financial Disclosure field and the Funding Information tab in your submission form. Note that the funders must be provided in the same order in both places as well. 
- State the initials, alongside each funding source, of each author to receive each grant.
- State what role the funders took in the study. If the funders had no role in your study, please state: “The funders had no role in study design, data collection and analysis, decision to publish, or preparation of the manuscript.”

**Response: We have clarified these points in the dedicated section.**

**Funding**

**“The research was partially funded by Toulouse University Hospital Center. XD was supported by grant from VITAM (Centre de recherche en santé durable, 2023-11), Laval University (Bourse d’Excellence de recherche sur le vieillissement, 05-2022) and Rotary Foundation (grant number : GG2575114). The funders had no role in study design, data collection and analysis, decision to publish, or preparation of the manuscript.”**

2) In your Data Availability Statement, please ensure you have provided all necessary access information in complete sentences. Access information should include the name of any data repositories used as well as digital object identifiers (DOIs), accession numbers or codes, or other persistent identifiers for all relevant data. If all data has been provided within your submission files, please use the statement "All relevant data is available within the manuscript and Supporting Information files." The Data Availability Statement will appear on the published version of your manuscript.

**Response:**

**I ensure that we provide all the necessary information.**

3) Please provide separate figure files in .tif or .eps format. For more information about figure files please see our guidelines:

<https://journals.plos.org/plosmedicine/s/figures>

**Response:**

**We changed their format and downloaded them as separate files.**

4) We have noticed that you have uploaded Supporting Information files, but you have not included a list of legends. Please add a full list of legends for your Supporting Information files after the references list.

**Response:**

**We added a list of legends in supplementary.**

5) Figure 2: Please confirm whether you drew the images / clip-art within the figure panels by hand. If you did not draw the images, please provide (a) a link to the source of the images or icons and their license / terms of use; or (b) written permission from the copyright holder to publish the images or icons under our CC BY 4.0 license. Alternatively, you may replace the images with open source alternatives. See these open source resources you may use to replace images / clip-art:
- <https://commons.wikimedia.org>
- <https://openclipart.org/>

**Response:**

**I confirm that we created this image. It is original for this article.**

**Reviewer #1:**

The manuscript Risk stratification for acute traumatic intracranial haemorrhage in older adults after a ground-level fall using head impact location describes a retrospective multicenter study on mTBI patients. The study has an interesting design and the question is important to the scientific community. To increase the impact of the paper and help clinicians to draw benefit from the data some modifications/explantations would be helpfull:

The number of "positive" CCTs is not very high; this counts even more since 15 patients had bleeding without clinical symptoms: so, from my perspective it is not easy to understand how the authors can have a sensitivity of 100% with their rule; it would be good to explain this difference, why was a CT done in these individuals?

**Response:**

**As we described in the methods section, the decision to prescribe a CT scan was left to the discretion of the emergency physician. This result confirms the hypothesis that among asymptomatic patients, the presence and location of the external impact may help identify those at risk of hemorrhagic injury. Among these 15 asymptomatic patients, all had sustained a forehead or scalp impact.**

Moreover, recently in many international guidelines the measurment of serum biomarkers such as GFAP or UCHL-1 were implemented. The authors have not used these markers, so it would be very good to discuss this topic also, because by the use of biomarkers the sensitivity could be improved significantly.

**Response:**

**Indeed, the primary objective of our research is to propose a risk stratification based on variables derived from clinical history and physical examination. This study therefore represents the first step before implementing biomarkers. We have added this perspective in the Discussion section.**

**Discussion**

**“We hypothesize that rule PIWI 1 could be used in patients with a clear history, while rule PIWI 2 could be applied in other cases. Moreover, based on this risk stratification, further studies could explore the use of biomarkers.”**

Beside these issues, the manuscriipt is very worthy to be published and it has a significant new information for the scientific community, so it will be cited frequently

**Reviewer #2:**

This manuscript aims to develop clinical decision rules (CDR's) for estimating the risk of significant intracranial hemorrhage in older patients presenting with mild TBI (GCS 13-15). Two CDR's were developed, each with 100% sensitivity and specificities ranging from 25 to 44%. Internal validation with bootstrapping showed good performance (AUC 0.79-0.84). Use of these CDR's would have permitted a reduction of 24 to 42% of number of CT scans performed. The study is of interest and relevant, given both the increasing number of older patients with TBI and the fact that existing CDR's have not been specifically developed for older patients. I have a number of comments and suggestions for consideration by the authors:

1.: I feel that the role of "head impact location" is over-emphasized by including it in the title. Table 3 shows that it is only ranked #5 in prognostic strength according to the odds ratio.

**Response:**

**We have therefore removed this from the title. In response to Reviewer 1’s comment, we believe it is important to emphasize this factor throughout the manuscript, as 15 of the 72 patients with hemorrhagic lesions were asymptomatic. It was only the presence and location of the impact that allowed us to identify the risk of hemorrhagic injury in these cases.**

2.: I object to the description of "minor TBI" for patients with a GCS of 13-15, and suggest to conform to the commonly used term of "mild TBI" - recognizing however that this term is debated.

**Response:**

**Indeed, this term is debated. In accordance with your comment, we have opted for the term "mild TBI" throughout the manuscript.**

3.: The Methods section should specifically mention that this was a prospective study.

**Response:**

**We added this in the method section.**

**Study design**

**“This multicentre prospective cohort study was conducted in five university-affiliated emergency departments located in France, Monaco, and Slovakia, with a combined annual emergency department attendance of approximately 352,000 visits.”**

4.: The inclusion of a second CRF entry by a different emergency physician is a strong point, but a weaker aspect is that this was only performed in 69 cases (Line 264). Why so few? Was that only performed in one or 2 selected sites?

**Response:**

**This inter-observer assessment was conducted across all sites but was subject to the constraints of emergency care. As mentioned in our methods section, "Wherever feasible, we asked the initial attending physician to have a second on-duty emergency physician independently complete a second data collection form, blinded to the initial collection, to calculate interobserver agreement." This step is indeed challenging to implement, as it requires a second physician who is blinded to the data collected by the first and available to perform this additional assessment. To our knowledge, there is no standard methodological approach for developing this type of rule that specifies how to determine the number of patients requiring double review. Upon reviewing similar studies that derived clinical decision rules, we found that the rate of double review in our study is comparable to others. For instance, the study by Perry et al., published in the BMJ in 2010 (Clinical decision rule to predict subarachnoid hemorrhage in patients with acute headache), involved double observation in only 103 out of the 1,999 included patients.**

5.: It is a bit surprising that only 49/72 patients with significant intracranial hemorrhage required hospitalization. Would that imply that these lesions were perhaps not so significant? Please expand and clarify.

**This result could indeed be confusing. The absence of hospitalization does not necessarily mean that the lesion is not significant. In fact, these patients are monitored in the emergency department without being formally admitted. Unfortunately, our data do not allow us to determine how long patients were monitored in the emergency department before being discharged. I suggest removing this sentence from the results, as we lack additional data to further detail this population. Therefore, we would retain only the previous sentence specifying the hospitalization rate for the entire cohort.**

**Results**

**In the overall cohort, 588 patients (36.3%) were hospitalized. ~~Among those with significant acute traumatic intracranial haemorrhage, 49 patients (68.1%) required hospitalization.~~**

6.: The Discussion would benefit from some comparison how your new CDR's perform relative to existing CDR's; although existing CDR's were not specifically developed for older people, it would be relevant to know if your CDR's performed better or not.

**Response:
In the Introduction, we detail recent rules developed for this population (CTHEAD, FALLS, FLORIDA). We considered integrating a comparison of the performance of these rules but ultimately decided against it for the following reasons:**

- **The CTHEAD rule excludes patients on anticoagulants, introducing a major risk of selection bias (45.1% of our cohort is on anticoagulants).**
- **To calculate the FLORIDA score, the mode of patient arrival (e.g., paramedics, relatives) must be known. Unfortunately, this information is not available in our cohort.**
- **The FALLS rule includes all elderly fall patients, whereas our study only includes patients with mild TBI. Additionally, the outcome measured in FALLS differs significantly from ours: FALLS considers patients with a lesion if they present with a hemorrhagic lesion within 42 days of the fall. In our PIWI study, patients with a lesion are defined as those presenting with a significant hemorrhagic lesion on the emergency CT scan.**

Minor:
1.: Abstract, line 13: Should "such injury broadly" be deleted?
2.: Intro, line 57: Delete the duplicate "but"
3.: Line 112: "judgment~" should be "judgement"
4.: Line 264: "pour" should be "in"
5.: Line 337: "specificites" should likely be "specifics'
6.: Line 385: "injur" should be "injury"

**Response:**

**We have corrected all of these errors.**

**Reviewer #3:**

This is an interesting study on the risk stratification for acute traumatic intracranial haemorrhage in older adults after a ground-level fall using head impact location. However, there are a few major issues needing attention.

1. Although the authors claimed they followed the TRIPOD statement, there is no external validations of the models at all. This is not acceptable for a complete clinical prediction model as external validation is a crucial part of the whole process.

**Response:**

**Indeed, in line with our objective, the purpose of this study is to derive a clinical decision rule. This derivation follows the TRIPOD recommendations. In accordance with the TRIPOD statement, we also performed internal validation using bootstrap. We are fully aware that this internal validation is not sufficient. This is why, at the end of our discussion, we propose that the next step will be the external validation of this clinical decision rule. Furthermore, we clearly state in the discussion that, at this stage of our research, the rule cannot yet be used in clinical practice and that external validation is necessary: "External validation of this rule is now necessary before it can be implemented in clinical practice, but these findings may already help identify high-risk patients."**

2. Sample size is relatively small. The 72 cases are the minimal requirement for such a model with 6 or 7 variables but definitely not ideal or good enough. For a reliable and robust model, more cases are needed.

**Response:**

**We have indeed added this point to the limitations section. Based on our preliminary data, the required sample size calculation indicated the need for 80 patients with the outcome of interest. Ultimately, we observed 72 patients with a lesion. However, the confidence intervals are relatively narrow, suggesting that the risk of overfitting in the multivariate model is quite low. Additionally, the stability of the model is further supported by the bootstrap results, which demonstrate that the model performs well.**

**Limitations**

**“…Regarding the statistical analysis, 72 patients had significant acute traumatic intracranial hemorrhage, compared to the 80 patients estimated during the sample size calculation. This lower-than-expected prevalence may increase the risk of overfitting in the multivariate models. »**

3. Variable selection. In table 3, we can see both 'Sign of basal skull fracture' and 'Headache' are not statistically significant, but both still appeared in the two derived rules later on as shown in Figure 2, which is inadequate.

**Response:**

**In accordance with standard methodological practices, we used two methods to identify predictors for inclusion in the final rule: logistic regression and recursive partitioning. As explained in the Methods section, combining these two approaches helps improve the final rule (““Predictors included in the rule were then identified through logistic regression and recursive partitioning. In line with standard methodology, combining these two techniques allows for correct patient classification regarding the outcome while minimizing the number of predictors.”)**

**This is why, although "headache" and "sign of basal skull fracture" were not significantly associated with the risk of lesions in logistic regression, their inclusion in the recursive partitioning allowed us to achieve a sensitivity of 100%. We have provided the details of the recursive partitioning process in the supplementary materials (S8 Figure. Recursive partitioning page 9-10).**

4. Not sure why two rules were developed. One should be fine and also depends on how good they perfrom in external validations.

**Response:**

**In line with our objectives, we aim to stratify the risk of hemorrhagic lesions using a rule adapted to the entire population of interest. This is why, as we explain in the Discussion section, we propose two rules: "to develop rules applicable specifically to our population of interest while offering a pragmatic approach to physicians for routine practice." From our perspective, keeping only one rule would be detrimental, as the PIWI 2 rule, though applicable in all situations, has low specificity. Conversely, the PIWI 1 rule, while highly specific, is only applicable to a subset of our population of interest. We agree with you that the external validation phase will provide an opportunity to evaluate the performance of each rule and determine the clinical situations in which one or the other may be best utilized.**

Table 2 is very much an ad hoc way to triage patients. The rules are only good at 'ruling out', but how practical and reliable especially for delayed bleeding after initial CT. For prediction models developed using logistica regression, we would expect to calculate a probability of intracranial haemorrhage using the variables, and then apply a cut-off to decide if it is a yes or no. So far, none is there.

**Response:**

**Recent literature regarding the risk of delayed hemorrhage in this population suggests that this risk is very low (reference: R. D. Shih et al., « Low Incidence of Delayed Intracranial Hemorrhage in Geriatric Emergency Department Patients on Preinjury Anticoagulation Presenting with Blunt Head Trauma », The Journal of Emergency Medicine). This is why we defined our primary outcome based on the emergency cerebral CT scan. Regarding the performance of these rules, their very high sensitivity indeed supports their role in "ruling out" conditions. Concerning the selection of variables in the final rule, we followed the standard methodology for developing such rules, which involves using two methods (logistic regression and recursive partitioning) to identify the minimum set of criteria needed to optimize the performance of the developed rule.**

5. Table 2 is not appropriate. Percentages should be collum wise rather than row wise.

**Response:**

**We have made this modification**

**Table 2: Interobserver agreement and univariate analysis for significant acute traumatic hemorrhage**

|  | No significant acute traumatic intracranial hemorrhage  (N = 1548) | Significant acute traumatic intracranial hemorrhage  (N = 72) | p-value | Kappa  (N = 69) |
| --- | --- | --- | --- | --- |
| Age, years | 84.6 (±8.5) | 83.8 (±8.4) | 0.45 | NA |
| Sex |  |  | 0.02 | NA |
| Male | 621 (40.1) | 39 (54.2) |  |  |
| Female | 927 (59.9) | 33 (45.8) |  |  |
| Cognitive impairment | 387 (25.0) | 25 (34.7) | 0.07 | NA |
| Frailty assessment |  |  | 0.07 |  |
| Robust (CFS* 1 – 3) | 528 (34.6) | 17 (25.8) |  |  |
| Frail (CFS 4 – 6) | 821 (53.9) | 45 (68.1) |  |  |
| Severely frail (CFS 7 – 9) | 175 (11.5) | 4 (6.1) |  |  |
| Antiplatelet medication | 539 (34.8) | 24 (33.3) | 0.8 | NA |
| Anticoagulant medication | 702 (45.3) | 29 (40.3) | 0.45 | NA |
| Precipitating factor for falls |  |  | 0.13 |  |
| Environmental | 880 (56.8) | 31 (43.1) |  | 0.88 |
| Not assessable | 381 (24.6) | 24 (33.3) |  | 0.76 |
| Dizziness | 145 (9.4) | 6 (8.3) |  | 0.63 |
| Syncope | 60 (3.9) | 4 (5.6) |  | 0.79 |
| Alcohol intoxication | 46 (3.0) | 5 (6.9) |  | 1.0 |
| Other | 36 (2.3) | 2 (2.8) |  | 0.68 |
| Post trauma symptoms |  |  |  |  |
| None | 857 (55.4) | 15 (20.1) | <0.001 | 0.72 |
| Acute confusion | 185 (12.0) | 24 (33.3) | <0.001 | 0.67 |
| Fall-related amnesia | 170 (11.0) | 10 (13.9) | 0.44 | 0.41 |
| Headache | 160 (10.3) | 23 (32.0) | 0.09 | 0.85 |
| Lost of consciousness | 118 (7.6) | 18 (25.0) | <0.001 | 0.63 |
| Vomiting ≥ 1 | 68 (4.4) | 15 (20.8) | <0.001 | 0.73 |
| Not assessable | 72 (4.6) | 1 (1.4) | 0.2 | 0.36 |
| Seizure | 14 (0.1) | 1 (1.4) | 0.67 | 0 |
| Physical examination |  |  |  |  |
| Glasgow Coma scale score below baseline | 134 (8.7) | 25 (34.7) | <0.001 | 0.63 |
| Signs of basal skull fracture | 56 (3.6) | 5 (6.9) | 0.14 | 0.67 |
| Pupil abnormalities | 43 (2.8) | 4 (5.6) | 0.17 | 0.71 |
| Focal neurological deficit | 20 (1.3) | 7 (9.7) | <0.001 | 0.68 |
| Visible head impact location |  |  | <0.001 | 0.88 |
| Scalp impact | 501 (32.4) | 41 (56.9) |  |  |
| Forehead impact | 499 (32.2) | 14 (19.4) |  |  |
| Facial impact | 172 (11.1) | 6 (8.4) |  |  |
| No visible impact | 376 (24.3) | 11 (15.3) |  |  |
| Visible head impact characteristics |  |  |  |  |
| Bruise | 385 (24.9) | 22 (30.6) | 0.27 | 0.81 |
| Subcutaneous hematoma | 446 (28.8) | 26 (36.1) | 0.18 | 0.76 |
| Wound requiring suture | 524 (33.9) | 20 (27.8) | 0.28 | 0.93 |

*CFS: Clinical Frailty Scale. NA=not applicable.

6. Overall, the study is more like an assiocation study finding risk factors of intracranial haemorrhage but short of a complete, robust and practical clinical prediction model.

**Response:**

**As indicated by the title of the article, the objective of this study is indeed to identify risk stratification for significant acute traumatic intracranial hemorrhage in older adults after a ground-level fall. We propose two rules specifically designed for this population to stratify this risk, offering physicians a pragmatic approach to evaluating it. However, before recommending its use, external validation is necessary.**

**General editorial requests:**
(Note: not all will apply to your paper, but please check each item carefully)

*	We ask every co-author listed on the manuscript to fill in a contributing author statement, making sure to declare all competing interests. If any of the co-authors have not filled in the statement, we will remind them to do so when the paper is revised. If all statements are not completed in a timely fashion this could hold up the re-review process. If new competing interests are declared later in the revision process, this may also hold up the submission. Should there be a problem getting one of your co-authors to fill in a statement we will be in contact. Please do not add or remove authors without first discussing this with the handling editor. You can see our competing interests policy here: <http://journals.plos.org/plosmedicine/s/competing-interests>.

**Response:**

**We have formatted the author contributions according to the standard requirements. Additionally, we have included a signed contributing author statement from each author.**

 Xavier Dubucs (XD) : Conceptualization, Methodology, Formal analysis, Investigation, Data curation, Writing – original draft, Writing – review & editing, Project administration.

 Cassandre Even (CE) : Formal analysis, Writing – original draft, Writing – review & editing, Project administration, Data curation

 Jemery Guenezan (JG) : Conceptualization, Methodology, Investigation, Writing – review & editing, Project administration, Supervision.

 Margaux Lagache (ML) : Formal analysis, Writing – original draft, Writing – review & editing, Project administration, Data curation

 Barbara Villoing (BV) : Conceptualization, Methodology, Investigation, Data curation, Writing – review & editing, Project administration, Supervision.

 Lorraine Pouzet (LP) : Formal analysis, Writing – original draft, Writing – review & editing, Project administration, Data curation

 Marián Sedlák (MS) : Conceptualization, Investigation, Data curation, Writing – review & editing, Project administration,

 Radoslav Morochovič (RM) : Project administration, Supervision.

 Yann-Érick Claessens (YC) : Project administration, Supervision.

 Valérie Boucher (VB) : Project administration, Writing – review & editing

 Sandrine Charpentier (SC) : Conceptualization, Methodology, Investigation, Data curation, Writing – review & editing, Project administration.

 Frédéric Balen (FB) : Conceptualization, Methodology, Writing – review & editing

 Éric Mercier (ÉM) : Conceptualization, Methodology, Investigation, Data curation, Writing – original draft, Writing – review & editing, Supervision.

 Marcel Émond (MÉ) : Conceptualization, Methodology, Formal analysis, Investigation, Data curation, Writing – review & editing, Supervision.

*	Please upload any figures associated with your paper as individual TIF or EPS files with 300dpi resolution at resubmission; please read our figure guidelines for more information on our requirements: <http://journals.plos.org/plosmedicine/s/figures>. While revising your submission, we strongly recommend that you use PLOS's NAAS tool (<https://ngplosjournals.pagemajik.ai/artanalysis>) to test your figure files. NAAS can convert your figure files to the TIFF file type and meet basic requirements (such as print size, resolution), or provide you with a report on issues that do not meet our requirements and that NAAS cannot fix.

**We have uploaded the figures in the correct format.**

After uploading your figures to PLOS's NAAS tool - <https://ngplosjournals.pagemajik.ai/artanalysis>, NAAS will process the files provided and display the results in the "Uploaded Files" section of the page as the processing is complete.
If the uploaded figures meet our requirements (or NAAS is able to fix the files to meet our requirements), the figure will be marked as "fixed" above. If NAAS is unable to fix the files, a red "failed" label will appear above.
When NAAS has confirmed that the figure files meet our requirements, please download the file via the download option, and include these NAAS processed figure files when submitting your revised manuscript.

**We have uploaded the figures in the correct format.**


*	Please ensure that the paper adheres to the PLOS Data Availability Policy (see <http://journals.plos.org/plosmedicine/s/data-availability>), which requires that all data underlying the study's findings be provided in a repository or as Supporting Information. For data residing with a third party, authors are required to provide instructions with contact information (web or email address) for obtaining the data. Please note that a study author cannot be the contact person for the data. PLOS journals do not allow statements supported by "data not shown" or "unpublished results." For such statements, authors must provide supporting data or cite public sources that include it.

**We have modified the dedicated chapter to align with your editorial guidelines.**

“The findings and de-identified datasets supporting this study will be available immediately following publication upon reasonable request to the Department of Clinical Research and Innovation (DRCI) at Toulouse University Hospital. A formal data request, a summary of the planned analysis, and a signed Data Transfer Agreement (DTA) will be required. Upon execution of the DTA, data will be shared via secure, password-protected electronic files without further technical support from the authors. All data-sharing procedures will strictly comply with the European Union’s General Data Protection Regulation (GDPR), as well as the policies defined by the relevant Institutional Review Boards and French national health data regulations”

*	Please ensure that the study is reported according to the TRIPOD AND STROBE guidelines and include the completed checklists as Supporting Information. When completing the checklist, please use section and paragraph numbers, rather than page numbers. Please add the following statement, or similar, to the Methods: "This study is reported as per [XXXX] guideline (S1 Checklist)."

**TRIPOD and SROBE checklist are provided in the appendix**

| **S10 TRIPOD Checklist** | **12** |
| --- | --- |
| **S11 STROBE Checklist** | **13** |

FORMATTING - GENERAL
*	Abstract: Please structure your abstract using the PLOS Medicine headings (Background, Methods and Findings, Conclusions). Please combine the Methods and Findings sections into one section.

**I have formatted the abstract in accordance with your editorial guidelines.**

*	At this stage, we ask that you include a short, non-technical Author Summary of your research to make findings accessible to a wide audience that includes both scientists and non-scientists. The Author Summary should immediately follow the Abstract in your revised manuscript. This text is subject to editorial change and should be distinct from the scientific abstract. Ideally each sub-heading should contain 2-3 single sentence, concise bullet points containing the most salient points from your study. In the final bullet point of 'What Do These Findings Mean?', please include the main limitations of the study in non-technical language. Please see our author guidelines for more information: <https://journals.plos.org/plosmedicine/s/revising-your-manuscript#loc-author-summary>.

**These points are included in our article.**

*	Please express the main results with 95% CIs as well as p values. When reporting p values please report as p<0.001 and where higher as the exact p value p=0.002, for example. Throughout, suggest reporting statistical information as follows to improve clarity for the reader "22% (95% CI [13%,28%]; p</=)". Please be sure to define all numerical values at first use.

*	Please include page numbers and line numbers in the manuscript file. Use continuous line numbers (do not restart the numbering on each page). 

*	Please cite the reference numbers in square brackets. Citations should precede punctuation.

**We have addressed each of these points**

FIGURES AND TABLES
*	Please provide titles and legends for all figures and tables (including those in Supporting Information files).

*	Please define all abbreviations used in each figure/table (including those in Supporting Information files).

*	Please consider avoiding the use of red and green in order to make your figure more accessible to those with color blindness.

**We have checked these points.**

SUPPLEMENTARY MATERIAL
*	Please note that supplementary material will be posted as supplied by the authors. Therefore, please amend it according to the relevant comments outlined here.

*	Please cite your Supporting Information as outlined here: <https://journals.plos.org/plosmedicine/s/supporting-information>

**We have checked these points.**

REFERENCES
*	PLOS uses the numbered citation (citation-sequence) method and first six authors, et al.

*	Please ensure that journal name abbreviations match those found in the National Center for Biotechnology Information (NCBI) databases (<http://www.ncbi.nlm.nih.gov/nlmcatalog/journals>), and are appropriately formatted and capitalised.

*	Where website addresses are cited, please include the complete URL and specify the date of access (e.g. [accessed: 12/06/2023]).

*	Please also see <https://journals.plos.org/plosmedicine/s/submission-guidelines#loc-references> for further details on reference formatting.

**We have checked these points.**

STUDY TYPE-SPECIFIC REQUESTS:


OBSERVATIONAL STUDIES

*	Abstract: Please include the study design, population and setting, number of participants, years during which the study took place (enrollment and follow up), length of follow up, and main outcome measures.

**We have addressed each of these points**

*	Please ensure that the study is reported according to the STROBE (or appropriate STOBE extension) guideline (available from: <https://www.equator-network.org/reporting-guidelines/strobe>) and include the completed STROBE (or STROBE extension) checklist as Supporting Information. Please add the following statement, or similar, to the Methods: "This study is reported as per the Strengthening the Reporting of Observational Studies in Epidemiology (STROBE) guideline (S1 Checklist)." When completing the checklist, please use section and paragraph numbers, rather than page numbers.

**We mention compliance with the STROBE guidelines in the methods section and provide the checklist in the supplementary materials**

*	[FOR POPULATION HEALTH/REGISTRY STUDIES] Please ensure that the study is reported according to the RECORD guideline (available from <https://www.record-statement.org>) and include the completed checklist as Supporting Information. Please add the following statement, or similar, to the Methods: "This study is reported as per the Reporting of Studies Conducted using Observational Routinely-Collected Data (RECORD) guideline (S1 Checklist)." When completing the checklist, please use section and paragraph numbers, rather than page numbers.

**N/A**

*	[FOR POPULATION HEALTH ESTIMATES] Please ensure that the study is reported according to the GATHER statement (available from <https://www.equator-network.org/reporting-guidelines/gather-statement>) and include the completed checklist as Supporting Information. Please add the following statement, or similar, to the Methods: "This study is reported as per the Guidelines for Accurate and Transparent Health Estimates Reporting (GATHER) statement (S1 Checklist)." When completing the checklist, please use section and paragraph numbers, rather than page numbers.

**N/A**

*	[FOR MEDIATION ANALYSES] We recommend that the study is reported according to the AGReMA statement (<https://agrema-statement.org/#:~:text=AGReMA%20is%20an%20evidence%2D%20and,randomised%20trials%20and%20observational%20studies>) and include the completed checklist as Supporting Information. Please add the following statement, or similar, to the Methods: "This study is reported as per the Guideline for Reporting Mediation Analyses (AGReMA) statement (S1 Checklist)." When completing the checklist, please use section and paragraph numbers, rather than page numbers. 

*	For all observational studies, in the manuscript text, please indicate: (1) the specific hypotheses you intended to test, (2) the analytical methods by which you planned to test them, (3) the analyses you actually performed, and (4) when reported analyses differ from those that were planned, transparent explanations for differences that affect the reliability of the study's results. If a reported analysis was performed based on an interesting but unanticipated pattern in the data, please be clear that the analysis was data driven.

**All of these data are present in our manuscript.**

*	Please state in the Methods section whether the study had a prospective protocol or analysis plan. If a prospective analysis plan (from your funding proposal, IRB or other ethics committee submission, study protocol, or other planning document written before analyzing the data) was used in designing the study, please include the relevant document(s) with your revised manuscript as a Supporting Information file to be published alongside your study and cite it in the Methods section. A legend for this file should be included at the end of your manuscript. If no such document exists, please make sure that the Methods section transparently describes when analyses were planned, and when/why any data-driven changes to analyses took place. Changes in the analysis, including those made in response to peer review comments, should be identified as such in the Methods section of the paper, with rationale.

**The entire analysis plan is described in the methods section.**

MODELLING STUDIES

The following list is derived from Geoffrey P Garnett, Simon Cousens, Timothy B Hallett, Richard Steketee, Neff Walker. Mathematical models in the evaluation of health programmes. (2011) Lancet DOI:10.1016/S0140-6736(10)61505-X:

*	If pertinent, please provide a diagram that shows the model structure, including how the natural history of the disease is represented, the process and determinants of disease acquisition, and how the putative intervention could affect the system.

**N/A**

*	Please provide a complete list of model parameters, including clear and precise descriptions of the meaning of each parameter, together with the values or ranges for each, with justification or the primary source cited and important caveats about the use of these values noted.

**N/A**

*	Please provide a clear statement about how the model was fitted to the data, including goodness-of-fit measure, the numerical algorithm used, which parameter varied, constraints imposed on parameter values, and starting conditions.

**N/A**

*	For uncertainty analyses, please state the sources of uncertainties quantified and not quantified [can include parameter, data, and model structure].

**N/A**

*	Please provide sensitivity analyses to identify which parameter values are most important in the model. Uncertainty estimates seek to derive a range of credible results on the basis of an exploration of the range of reasonable parameter values. The choice of method should be presented and justified.

**N/A**

*	Please discuss the scientific rationale for the choice of model structure and identify points where this choice could influence conclusions drawn. Please also describe the strength of the scientific basis underlying the key model assumptions.

**N/A**

*	For studies that develop a prediction model or evaluate its performance, please ensure that the study is reported according to the TRIPOD statement (<https://www.equator-network.org/reporting-guidelines/tripod-statement>) and include the completed checklist as Supporting Information. Please add the following statement, or similar, to the Methods: "This study is reported as per the Transparent Reporting of a Multivariable Prediction Model for Individual Prognosis Or Diagnosis (TRIPOD) statement (S1 Checklist)." For studies using machine learning, please use the TRIPOD-AI checklist. When completing the checklist, please use section and paragraph numbers, rather than page numbers.

**We mention compliance with the TRIPOD statement in the methods section and provide the checklist in the supplementary materials.**
