# Supplementary material for: Risk stratification for significant acute traumatic intracranial hemorrhage in older adults after a ground-level fall: A prospective multicentre cohort study
Source: PLoS Med. 2026 Aug 10;23(8):e1004935. doi: 10.1371/journal.pmed.1004935 (PMC13456197; doi:10.1371/journal.pmed.1004935)
Supplement: Supplementary file 14 [file pmed.1004935.s015.docx]

June 14th, 2026

To Katarzyna Starowicz, Associate Editor
Plos Medicine

Ref.:  PMEDICINE-D-26-00283R1
Title: Risk stratification for significant acute traumatic intracranial hemorrhage in older adults after a ground-level fall: A prospective multicentre cohort study

Dear Associate Editor,

Thank you for offering us the opportunity to revise our manuscript “Risk stratification for significant acute traumatic intracranial hemorrhage in older adults after a ground-level fall: A prospective multicentre cohort study”. We have revised the paper according to the Reviewer’s comments and Journal Requirements. In this letter, we address the changes made point by point.

On behalf of all authors,

Xavier Dubucs

Comments from the reviewers: 

**Reviewer #1:** Recommendation: Accept after minor revisions
This revised manuscript is substantially improved and addresses the prior reviewer concerns adequately. The study is clinically important, prospective, multicentre, and focused on a high-volume population in which CT overuse is a relevant problem. The derived PIWI rules are clearly presented and show 100% sensitivity with potential CT reductions of 41.7% and 24.2%, respectively, although external validation remains necessary before clinical implementation.

Minor points to address before acceptance:
*	The abstract still contains a residual wording error in the highlighted version: "following a ground-level fall and who underwent a computed tomography scan, such injury broadly." This phrase should be deleted.

**We apologize for this oversight; the previous version of the abstract had not been removed.**

*	Table 4 contains a typographical error: "95% Conficence Interval" should be "95% Confidence Interval."

**Done.**

*	The conclusion should more strongly state that the rules are not yet ready for implementation without independent external validation.

**Please find our new proposal below :**

**« Such rules need to be validated externally and independently before being proposed for implementation in clinical practice. Nevertheless, these rules may already be helpful in identifying high-risk patients. »**

*	The Author Summary should include the main limitations in plain language, particularly the absence of external validation and the relatively small number of outcome events.

**The following text has been added to this section:**

**Before being proposed for implementation in clinical practice, these rules need to be validated in other healthcare settings using large cohorts with a large number of outcome events.**

Overall, the manuscript is suitable for publication once these minor editorial and clarity issues are corrected.

**Reviewer #2:**

This manuscript is a revision of a previous submission. The authors have addressed most reviewer comments appropriately.

I am, however, not happy with their response to my (Reviewer 2) previous comment # 5. The authors have now deleted the sentence which I queried " Among those with significant acute intracranial haemorrhage, 49 patients (68.1%) required hospitalization". This would appear "an easy way out", and may be perceived as selective reporting. I suggest this sentence is re-inserted. Perhaps you could add a line stating something like (if correct) "Those not requiring admission to hospital received appropriate observation in the ER for longer periods of time, and were often discharged only after a second CT scan showing no progression". Might you know how many received a second CT Scan?

**Unfortunately, we do not know which patients received a second CT scan. We propose modifying the sentence as follows:**

**“Among those with significant acute traumatic intracranial hemorrhage, 49 patients (68.1%) required hospitalization, while the remaining patients underwent observation in the ED.”**

Minor comments:
1.	At various places in the manuscript and in the tables you report on "Reduced GCS from baseline" How did you define "baseline" Was this the presumed pre-injury GCS or the GCS on presentation?

**We have added this clarification to the Methods section**

**“The baseline Glasgow coma scale score was presumed from the patient's pre-injury status, based on interviews with the patient or witnesses”**

2.	Please check the formatting of the References, in particular to the format of listing 6 authors et al.

**Done**

**Reviewer #3**: Thanks authors for their effort to clarify my concerns. It's mostly fine as a risk stratification rule (or rules). I am satisfied with the response and revision. No further issues needing attention.

**EDITORIAL REQUESTS:**

* Please confirm that your title complies with PLOS Medicine's style. Your title must be nondeclarative and not a question. It should begin with main concept if possible. "Effect of" should be used only if causality can be inferred, i.e., for an RCT. Please place the study design ("A randomized controlled trial," "A retrospective study," "A modelling study," etc.) in the subtitle (ie, after a colon).

**We have corrected the formatting by capitalizing the 'A'.**

* Please confirm that your abstract complies with our requirements, including format (three sections: Background, Methods and Findings, and Conclusions) and providing all the information relevant to this study type <https://journals.plos.org/plosmedicine/s/submission-guidelines#loc-abstract>

**Done.**

* Please ensure that the Introduction ends with a clear description of the study question or hypothesis.

**Done.**

* Please ensure that all abbreviations are defined at first use throughout the text.

**Check.**

* Please confirm that all numbers presented in the abstract are present and identical to numbers presented in the main manuscript text.

**Check.**

* Please disclose any use of AI tools and technologies to the study or article content at the end of the Methods. Please specify the name(s) and version(s) of any tool(s) used and a description of their use, including how the AI-assisted or generated content was evaluated by authors. Please also include a statement specifying who retains responsibility for the final manuscript. Please see our guidelines for more information: <https://journals.plos.org/plosmedicine/s/ethical-publishing-practice#loc-artificial-intelligence-tools-and-technologies>.

**Check.**

* Please review your text for claims of novelty or primacy (e.g. 'for the first time') and remove this language. In addition, please check that any use of statistical terms (such as trend or significant) are supported by the data, and if not please remove them.

**Check**

* Please remove the 'conclusions' subheading from the discussion. Please also remove any other subheadings from the discussion.

**Done**

* Statistical reporting: Please revise throughout the manuscript, including tables and figures.
- Please report statistical information as follows to improve clarity for the reader ""22% (95% CI [13,28]; p</=)"".

**Done. This has been applied throughout the text and the figure.**

- Please separate upper and lower bounds with commas instead of hyphens as the latter can be confused with reporting of negative values.

**Done**

- Please repeat statistical definitions (HR, CI etc.) for each set of parentheses.

**We have standardized the presentation of the results across the text, tables, and figures, in accordance with the format suggested in your previous comment: « 22% (95% CI [13,28])**

* In the last sentence of the Abstract Methods and Findings section, please describe the main limitation(s) of the study's methodology.

**We added two sentence at the end of this section :**

**“Because we only included patients who underwent a head CT scan during their emergency department stay, the potential for selection bias must be considered. Additionally, a risk of misclassification bias exists because we did not perform a centralized independent review of the CT scans.”**

* In the abstract, please include the important dependent variables that are adjusted for in the analyses.

**We added:**

**Using logistic regression, associations between independent variables and the outcome were adjusted for age, antithrombotic medication (antiplatelet or anticoagulant), and precipitating factors for the fall.**

* In the author summary, please revise formatting and ensure you use bullet points.

**In line with Reviewer 1's comments, we have also improved this section.**

* In the author summary, in the final bullet point of 'What Do These Findings Mean?', please include the main limitations of the study in non-technical language.

**We added this sentence :**

**« Because we only included emergency department patients who underwent a head scan, and did not have those scans double-checked by a central team, there is a risk our results could be skewed or misclassified »**

* The funding statement should include: specific grant numbers, initials of authors who received each award, URLs to sponsors’ websites. Also, please state whether any sponsors or funders (other than the named authors) played any role in study design, data collection and analysis, the decision to publish, or preparation of the manuscript. If they had no role in the research, include this sentence: “The funders had no role in study design, data collection and analysis, 
decision to publish, or preparation of the manuscript.”

**Done**

* PLOS Medicine requires that the de-identified data underlying the specific results in a published article be made available, without restrictions on access, in a public repository or as Supporting Information at the time of article publication, provided it is legal and ethical to do so. Please see the policy at 
<http://journals.plos.org/plosmedicine/s/data-availability>  and FAQs at <http://journals.plos.org/plosmedicine/s/data-availability#loc-faqs-for-data-policy>  "

**Check**

* PLOS defines the “minimal data set” to consist of the data set used to reach the conclusions drawn in the manuscript with related metadata and methods, and any additional data required to replicate the reported study findings in their entirety. Authors do not need to submit their entire data set, or the raw data collected during an investigation. Please submit the following data:
The values behind the means, standard deviations and other measures reported;
The values used to build graphs;
The points extracted from images for analysis."

**Done. This is available in the Supporting Information**

* The Data Availability Statement (DAS) requires revision. For each data source used in your study: 
a) If the data are freely or publicly available, note this and state the location of the data: within the paper, in Supporting Information files, or in a public repository (include the DOI or accession number).
b) If the data are owned by a third party but freely available upon request, please note this and state the owner of the data set and contact information for data requests (web or email address). Note that a study author cannot be the contact person for the data.
c) If the data are not freely available, please describe briefly the ethical, legal, or contractual restriction that prevents you from sharing it. Please also include an appropriate contact (web or email address) for inquiries (again, this cannot be a study author).

**This corresponds to situation b). The data are owned by a third party (the Department of Clinical Research and Innovation [DRCI] at Toulouse University Hospital). We have added their contact email address to the Data Sharing section**

* For studies in which a novel model is central to the manuscript's findings, as is the case here, authors are responsible for providing the source code needed to replicate the study's findings in a repository (such as GitHub, SourceForge or Bitbucket) or a cloud computing service (such as Code Ocean).  Please include a citable digital object identifier (DOI) or other persistent identifier of the code (such as through linkage with Zenodo). Protection of authors’ intellectual property will not be cause for exception. Please explain in the manuscript’s Data Availability Statement how readers can access the shared code.

**N/A**

* Thank you for agreeing to make your data available. At this time, please provide the link to the data repository and accession numbers required for access.

**Done**

* Please include the statement on code availability in the data availability statement. Please see guidance here:<https://journals.plos.org/plosmedicine/s/code-sharing-guidance#loc-data-availability-statement-examples>

**Done**

* Please provide the name(s) of the institutional review board(s) that provided ethical approval.

**We added the name of this board  in this section :**

**“The institutional scientific board of Toulouse University Hospital’s Department of Clinical Research and Innovation approved this protocol.”**

* Please revise for use of patient-centered language. Please note that patient-centered language is constructed with the use of post-modified nouns (e.g. 'patients with psoriasis’ (or similar) instead of ‘psoriasis patients’) putting the person first in the sentence structure.

**Check**

* Please specify whether informed consent was written or oral. Please ensure that the research complies with the PLOS policy in full: <https://journals.plos.org/plosmedicine/s/human-subjects-research#loc-patient-privacy-and-informed-consent-for-publication>

**As mentionned in the methods section, “… Participants provided oral informed consent to participate in the study and received an information sheet explaining the research”.**

* Please provide titles and legends for all figures and tables (including those in Supporting Information files). Please define all acronyms used in each figure or table in its corresponding legend.

**Done in the manuscript and supporting information files.**

* When a p value is given, please specify the statistical test used to determine it in the legend.

**Done**

* Please consider avoiding the use of red and green in order to make your figure more accessible

**We have updated the color palette to enhance readability**

* Please provide the unadjusted comparisons as well as the adjusted comparisons in all relevant Tables

**Done.**

* Please specify the variables controlled for in all relevant Tables

**Done. This information was added to Table 3**

SUPPORTING INFORMATION:

* We have noticed that you have uploaded a list of legend in Supporting Information file. Please add a full list of legends for your Supporting Information files after the references list.

**Done**

* We do recommend upload each supporting information file separately. You may use almost any description as the item name of your supporting information as long as it contains an "S" and number. For example, “S1 Appendix” and “S2 Appendix,” “S1 Table” and “S2 Table,” and so forth.

**Done**

* Use whole numbers when naming your supporting information files. Combine separate parts (e.g., S1A and S1B Table) into one file (e.g. S1 Table) or rename with whole numbers (e.g., S1 and S2 Table).

**Done**

* Match the names of your supporting information files with the supporting information captions within your manuscript. For example, a PDF file for “S2 Fig.” must be named “S2_fig.pdf”.

**Done**

* The supporting information name and number are required in a caption, and we highly recommend including a one-line title as well. You may also include a legend in your caption, but it is not required. Format your supporting information captions as follows:

 S1 Text. Title is strongly recommended. Legend is optional.

**Done**

* In the published article, supporting information files are accessed only through a hyperlink attached to the captions. For this reason, you must list captions at the end of your manuscript file. You may include a caption within the supporting information file itself, as long as that caption is also provided in the manuscript file. Do not submit a separate caption file.

**Done**

* We do recommend that STROBE and TRIPOD Checklists are listed as S1 Checklist, S2 Checklist respectively.

**Done**

* Please revise your references to supporting information in the manuscript main text after implementing changes

**Done**

* Please include IRB approval as "Other" type of document in your resubmission as this document is for editorial purposes only

**Done**

* Please include The Study Protocol in English

**Done**
