# Supplementary material for: Risk stratification for significant acute traumatic intracranial hemorrhage in older adults after a ground-level fall: A prospective multicentre cohort study
Source: PLoS Med. 2026 Aug 10;23(8):e1004935. doi: 10.1371/journal.pmed.1004935 (PMC13456197; doi:10.1371/journal.pmed.1004935)
Supplement: Peer review history

# Editor report: Decision Letter 0

**DOI:** 10.1371/journal.pmed.1004935.r001

**Authors:** Katarzyna Starowicz

23 Jan 2026

Dear Dr Dubucs,

Thank you for submitting your manuscript entitled "Risk stratification for acute traumatic intracranial haemorrhage in older adults after a ground-level fall using head impact location" for consideration by PLOS Medicine.

Your manuscript has now been evaluated by the PLOS Medicine editorial staff, and I am writing to let you know that we would like to send your submission out for external peer review.

However, before we can send your manuscript to reviewers, we need you to complete your submission by providing the metadata that is required for full assessment. To this end, please login to Editorial Manager where you will find the paper in the 'Submissions Needing Revisions' folder on your homepage. Please click 'Revise Submission' from the Action Links and complete all additional questions in the submission questionnaire.

Additionally, as you mentioned in your manuscript that you followed TRIPOD and STROBE guidelines in your study, it might be useful for reviewers that you:

* Include the completed TRIPOD checklist as Supporting Information. Please add the following statement, or similar, to the Methods: "This study is reported as per the Transparent Reporting of a Multivariable Prediction Model for Individual Prognosis Or Diagnosis (TRIPOD) statement (S1 Checklist)." For studies using machine learning, please use the TRIPOD-AI checklist. When completing the checklist, please use section and paragraph numbers, rather than page numbers.

* Include the completed STROBE (or STROBE extension) checklist as Supporting Information. Please add the following statement, or similar, to the Methods: "This study is reported as per the Strengthening the Reporting of Observational Studies in Epidemiology (STROBE) guideline (S1 Checklist)." When completing the checklist, please use section and paragraph numbers, rather than page numbers.

For clinical studies, please upload a copy of your trial study protocol as a supporting information file. The study protocol should be the version submitted for approval to the institutional review board or ethics committee, should include any amendments to the study protocol, as well as the date of their approval by the institutional review or ethics committee. Please also detail any deviations from the study protocol in the Methods section of your manuscript. The editors will consider the protocol and study conduct prior to a final decision for external review.

Please re-submit your manuscript within two working days, i.e. by Jan 27 2026 11:59PM.

Login to Editorial Manager here: https://www.editorialmanager.com/pmedicine

Once your full submission is complete, your paper will undergo a series of checks in preparation for peer review. Once your manuscript has passed all checks it will be sent out for review.

Feel free to email us at plosmedicine@plos.org if you have any queries relating to your submission.

Kind regards,

Katarzyna Starowicz, PhD

Associate Editor

PLOS Medicine

kstarowicz@plos.org

# Peer review: Decision Letter 1

**DOI:** 10.1371/journal.pmed.1004935.r002

**Authors:** Katarzyna Starowicz

4 Mar 2026

Dear Dr Dubucs,

Many thanks for submitting your manuscript "Risk stratification for acute traumatic intracranial haemorrhage in older adults after a ground-level fall using head impact location" (PMEDICINE-D-26-00283R1) to PLOS Medicine. The paper has been reviewed by subject experts and a statistician; their comments are included below and can also be accessed here: [LINK]

As you will see, the reviewers finds your study interesting however, they raised concerns regarding certain methodological and analytical aspects of your study, particularly the need for external validation to enhance the impact of the manuscript and demonstrate the clinical utility of your model.

We were wondering if they are any existing independent clinical datasets you can use for external validation? If suitable data are accessible, could you incorporate an external validation analysis in the revised manuscript? Additionally, comparison of your model with existing clinical decision rules would provide important context regarding performance and add clinical value.

After discussing the paper with the editorial team and an academic editor with relevant expertise, I'm pleased to invite you to revise the paper in response to the reviewers' comments. We plan to send the revised paper to some or all of the original reviewers, and we cannot provide any guarantees at this stage regarding publication.

In addition to these revisions, you may need to complete some formatting changes, which you will receive in a follow up email. A member of our team will be in touch with a set of requests shortly. If you do not receive a separate email within a few days, please assume that checks have been completed, and no additional changes are required.

When you upload your revision, please include a point-by-point response that addresses all of the reviewer and editorial points, indicating the changes made in the manuscript and either an excerpt of the revised text or the location (eg: page and line number) where each change can be found. Please also be sure to check the general editorial comments at the end of this letter and include these in your point-by-point response. When you resubmit your paper, please include a clean version of the paper as the main article file and a version with changes tracked as a marked-up manuscript. It may also be helpful to check the guidelines for revised papers at http://journals.plos.org/plosmedicine/s/revising-your-manuscript for any that apply to your paper.

We ask that you submit your revision by Mar 25 2026 11:59PM. However, if this deadline is not feasible, please contact me by email, and we can discuss a suitable alternative.

Don't hesitate to contact me directly with any questions (kstarowicz@plos.org).

Best regards,

Katarzyna

Katarzyna Starowicz, PhD

Associate Editor

PLOS Medicine

kstarowicz@plos.org

-----------------------------------------------------------

Comments from the reviewers:

Reviewer #1: The manuscript Risk stratification for acute traumatic intracranial haemorrhage in older adults after a ground-level fall using head impact location describes a retrospective multicenter study on mTBI patients. The study has an interesting design and the question is important to the scientific community. To increase the impact of the paper and help clinicians to draw benefit from the data some modifications/explantations would be helpfull:

The number of "positive" CCTs is not very high; this counts even more since 15 patients had bleeding without clinical symptoms: so, from my perspective it is not easy to understand how theauthors can have a sensitivity of 100% with their rule; it would be good to explain this difference, why was a CT done in these individuals? Moreover, recently in many international guidelines the measurment of serum biomarkers such as GFAP or UCHL-1 were implemented. The authors have not used these markers, so it would be very good to discuss this topic also, because by the use of biomarkers the sensitivity could be improved significantly. Beside these issues, the manuscriipt is very worthy to be published and it has a significant new information for the scientific community, so it will be cited frequently

Reviewer #2: This manuscript aims to develop clinical decision rules (CDR's) for estimating the risk of significant intracranial hemorrhage in older patients presenting with mild TBI (GCS 13-15). Two CDR's were developed, each with 100% sensitivity and specificities ranging from 25 to 44%. Internal validation with bootstrapping showed good performance (AUC 0.79-0.84). Use of these CDR's would have permitted a reduction of 24 to 42% of number of CT scans performed. The study is of interest and relevant, given both the increasing number of older patients with TBI and the fact that existing CDR's have not been specifically developed for older patients. I have a number of comments and suggestions for consideration by the authors:

1.: I feel that the role of "head impact location" is over-emphasized by including it in the title. Table 3 shows that it is only ranked #5 in prognostic strength according to the odds ratio.

2.: I object to the description of "minor TBI" for patients with a GCS of 13-15, and suggest to conform to the commonly used term of "mild TBI" - recognizing however that this term is debated.

3.: The Methods section should specifically mention that this was a prospective study.

4.: The inclusion of a second CRF entry by a different emergency physician is a strong point, but a weaker aspect is that this was only performed in 69 cases (Line 264). Why so few? Was that only performed in one or 2 selected sites?

5.: It is a bit surprising that only 49/72 patients with significant intracranial hemorrhage required hospitalization. Would that imply that these lesions were perhaps not so significant? Please expand and clarify.

6.: The Discussion would benefit from some comparison how your new CDR's perform relative to existing CDR's; although existing CDR's were not specifically developed for older people, it would be relevant to know if your CDR's performed better or not.

Minor:

1.: Abstract, line 13: Should "such injury broadly" be deleted?

2.: Intro, line 57: Delete the duplicate "but"

3.: Line 112: "judgment~" should be "judgement"

4.: Line 264: "pour" should be "in"

5.: Line 337: "specificites" should likely be "specifics'

6.: Line 385: "injur" should be "injury"

Reviewer #3: This is an interesting study on the risk stratification for acute traumatic intracranial haemorrhage in older adults after a ground-level fall using head impact location. However, there are a few major issues needing attention.

1. Although the authors claimed they followed the TRIPOD statement, there is no external validations of the models at all. This is not acceptable for a complete clinical prediction model as external validation is a crucial part of the whole process.

2. Sample size is relatively small. The 72 cases are the minimal requirement for such a model with 6 or 7 variables but definitely not ideal or good enough. For a reliable and robust model, more cases are needed.

3. Variable selection. In table 3, we can see both 'Sign of basal skull fracture' and 'Headache' are not statistically significant, but both still appeared in the two derived rules later on as shown in Figure 2, which is inadequate.

4. Not sure why two rules were developed. One should be fine and also depends on how good they perfrom in external validations. Table 2 is very much an ad hoc way to triage patients. The rules are only good at 'ruling out', but how practical and reliable especially for delayed bleeding after initial CT. For prediction models developed using logistica regression, we would expect to calculate a probability of intracranial haemorrhage using the variables, and then apply a cut-off to decide if it is a yes or no. So far, none is there.

5. Table 2 is not appropriate. Percentages should be collum wise rather than row wise.

6. Overall, the study is more like an assiocation study finding risk factors of intracranial haemorrhage but short of a complete, robust and practical clinical prediction model.

Any attachments provided with reviews can be seen via the following link: [LINK]

--------------------------------------------------------- ---

General editorial requests:

(Note: not all will apply to your paper, but please check each item carefully)

*We ask every co-author listed on the manuscript to fill in a contributing author statement, making sure to declare all competing interests. If any of the co-authors have not filled in the statement, we will remind them to do so when the paper is revised. If all statements are not completed in a timely fashion this could hold up the re-review process. If new competing interests are declared later in the revision process, this may also hold up the submission. Should there be a problem getting one of your co-authors to fill in a statement we will be in contact. Please do not add or remove authors without first discussing this with the handling editor. You can see our competing interests policy here: http://journals.plos.org/plosmedicine/s/competing-interests.

*Please upload any figures associated with your paper as individual TIF or EPS files with 300dpi resolution at resubmission; please read our figure guidelines for more information on our requirements: http://journals.plos.org/plosmedicine/s/figures. While revising your submission, we strongly recommend that you use PLOS's NAAS tool (https://ngplosjournals.pagemajik.ai/artanalysis) to test your figure files. NAAS can convert your figure files to the TIFF file type and meet basic requirements (such as print size, resolution), or provide you with a report on issues that do not meet our requirements and that NAAS cannot fix.

After uploading your figures to PLOS's NAAS tool - https://ngplosjournals.pagemajik.ai/artanalysis, NAAS will process the files provided and display the results in the "Uploaded Files" section of the page as the processing is complete.

If the uploaded figures meet our requirements (or NAAS is able to fix the files to meet our requirements), the figure will be marked as "fixed" above. If NAAS is unable to fix the files, a red "failed" label will appear above.

When NAAS has confirmed that the figure files meet our requirements, please download the file via the download option, and include these NAAS processed figure files when submitting your revised manuscript.

*Please ensure that the paper adheres to the PLOS Data Availability Policy (see http://journals.plos.org/plosmedicine/s/data-availability), which requires that all data underlying the study's findings be provided in a repository or as Supporting Information. For data residing with a third party, authors are required to provide instructions with contact information (web or email address) for obtaining the data. Please note that a study author cannot be the contact person for the data. PLOS journals do not allow statements supported by "data not shown" or "unpublished results." For such statements, authors must provide supporting data or cite public sources that include it.

*We expect all researchers with submissions to PLOS in which author-generated code underpins the findings in the manuscript to make all author-generated code available without restrictions upon publication of the work. In cases where code is central to the manuscript, we may require the code to be made available as a condition of publication. Authors are responsible for ensuring that the code is reusable and well documented. Please make any custom code available, either as part of your data deposition or as a supplementary file. Please add a sentence to your data availability statement regarding any code used in the study, e.g. "The code used in the analysis is available from Github [URL] and archived in Zenodo [DOI link]" Please review our guidelines at https://journals.plos.org/plosmedicine/s/materials-software-and-code-sharing and ensure that your code is shared in a way that follows best practice and facilitates reproducibility and reuse. Because Github depositions can be readily changed or deleted, we encourage you to make a permanent DOI'd copy (e.g. in Zenodo) and provide the URL.

*Please ensure that the study is reported according to the TRIPOD AND STROBE guidelines and include the completed checklists as Supporting Information. When completing the checklist, please use section and paragraph numbers, rather than page numbers. Please add the following statement, or similar, to the Methods: "This study is reported as per [XXXX] guideline (S1 Checklist)."

FORMATTING - GENERAL

*Abstract: Please structure your abstract using the PLOS Medicine headings (Background, Methods and Findings, Conclusions). Please combine the Methods and Findings sections into one section.

*At this stage, we ask that you include a short, non-technical Author Summary of your research to make findings accessible to a wide audience that includes both scientists and non-scientists. The Author Summary should immediately follow the Abstract in your revised manuscript. This text is subject to editorial change and should be distinct from the scientific abstract. Ideally each sub-heading should contain 2-3 single sentence, concise bullet points containing the most salient points from your study. In the final bullet point of 'What Do These Findings Mean?', please include the main limitations of the study in non-technical language. Please see our author guidelines for more information: https://journals.plos.org/plosmedicine/s/revising-your-manuscript#loc-author-summary.

*Please express the main results with 95% CIs as well as p values. When reporting p values please report as p<0.001 and where higher as the exact p value p=0.002, for example. Throughout, suggest reporting statistical information as follows to improve clarity for the reader "22% (95% CI [13%,28%]; p</=)". Please be sure to define all numerical values at first use.

*Please include page numbers and line numbers in the manuscript file. Use continuous line numbers (do not restart the numbering on each page).

*Please cite the reference numbers in square brackets. Citations should precede punctuation.

FIGURES AND TABLES

*Please provide titles and legends for all figures and tables (including those in Supporting Information files).

*Please define all abbreviations used in each figure/table (including those in Supporting Information files).

*Please consider avoiding the use of red and green in order to make your figure more accessible to those with color blindness.

SUPPLEMENTARY MATERIAL

*Please note that supplementary material will be posted as supplied by the authors. Therefore, please amend it according to the relevant comments outlined here.

*Please cite your Supporting Information as outlined here: https://journals.plos.org/plosmedicine/s/supporting-information

REFERENCES

*PLOS uses the numbered citation (citation-sequence) method and first six authors, et al.

*Please ensure that journal name abbreviations match those found in the National Center for Biotechnology Information (NCBI) databases (http://www.ncbi.nlm.nih.gov/nlmcatalog/journals), and are appropriately formatted and capitalised.

*Where website addresses are cited, please include the complete URL and specify the date of access (e.g. [accessed: 12/06/2023]).

*Please also see https://journals.plos.org/plosmedicine/s/submission-guidelines#loc-references for further details on reference formatting.

STUDY TYPE-SPECIFIC REQUESTS:

OBSERVATIONAL STUDIES

*Abstract: Please include the study design, population and setting, number of participants, years during which the study took place (enrollment and follow up), length of follow up, and main outcome measures.

*Please ensure that the study is reported according to the STROBE (or appropriate STOBE extension) guideline (available from: https://www.equator-network.org/reporting-guidelines/strobe) and include the completed STROBE (or STROBE extension) checklist as Supporting Information. Please add the following statement, or similar, to the Methods: "This study is reported as per the Strengthening the Reporting of Observational Studies in Epidemiology (STROBE) guideline (S1 Checklist)." When completing the checklist, please use section and paragraph numbers, rather than page numbers.

*[FOR POPULATION HEALTH/REGISTRY STUDIES] Please ensure that the study is reported according to the RECORD guideline (available from https://www.record-statement.org) and include the completed checklist as Supporting Information. Please add the following statement, or similar, to the Methods: "This study is reported as per the Reporting of Studies Conducted using Observational Routinely-Collected Data (RECORD) guideline (S1 Checklist)." When completing the checklist, please use section and paragraph numbers, rather than page numbers.

*[FOR POPULATION HEALTH ESTIMATES] Please ensure that the study is reported according to the GATHER statement (available from https://www.equator-network.org/reporting-guidelines/gather-statement) and include the completed checklist as Supporting Information. Please add the following statement, or similar, to the Methods: "This study is reported as per the Guidelines for Accurate and Transparent Health Estimates Reporting (GATHER) statement (S1 Checklist)." When completing the checklist, please use section and paragraph numbers, rather than page numbers.

*[FOR MEDIATION ANALYSES] We recommend that the study is reported according to the AGReMA statement (https://agrema-statement.org/#:~:text=AGReMA%20is%20an%20evidence%2D%20and,randomised%20trials%20and%20observational%20studies) and include the completed checklist as Supporting Information. Please add the following statement, or similar, to the Methods: "This study is reported as per the Guideline for Reporting Mediation Analyses (AGReMA) statement (S1 Checklist)." When completing the checklist, please use section and paragraph numbers, rather than page numbers.

*For all observational studies, in the manuscript text, please indicate: (1) the specific hypotheses you intended to test, (2) the analytical methods by which you planned to test them, (3) the analyses you actually performed, and (4) when reported analyses differ from those that were planned, transparent explanations for differences that affect the reliability of the study's results. If a reported analysis was performed based on an interesting but unanticipated pattern in the data, please be clear that the analysis was data driven.

*Please state in the Methods section whether the study had a prospective protocol or analysis plan. If a prospective analysis plan (from your funding proposal, IRB or other ethics committee submission, study protocol, or other planning document written before analyzing the data) was used in designing the study, please include the relevant document(s) with your revised manuscript as a Supporting Information file to be published alongside your study and cite it in the Methods section. A legend for this file should be included at the end of your manuscript. If no such document exists, please make sure that the Methods section transparently describes when analyses were planned, and when/why any data-driven changes to analyses took place. Changes in the analysis, including those made in response to peer review comments, should be identified as such in the Methods section of the paper, with rationale.

MODELLING STUDIES

The following list is derived from Geoffrey P Garnett, Simon Cousens, Timothy B Hallett, Richard Steketee, Neff Walker. Mathematical models in the evaluation of health programmes. (2011) Lancet DOI:10.1016/S0140-6736(10)61505-X:

*If pertinent, please provide a diagram that shows the model structure, including how the natural history of the disease is represented, the process and determinants of disease acquisition, and how the putative intervention could affect the system.

*Please provide a complete list of model parameters, including clear and precise descriptions of the meaning of each parameter, together with the values or ranges for each, with justification or the primary source cited and important caveats about the use of these values noted.

*Please provide a clear statement about how the model was fitted to the data, including goodness-of-fit measure, the numerical algorithm used, which parameter varied, constraints imposed on parameter values, and starting conditions.

*For uncertainty analyses, please state the sources of uncertainties quantified and not quantified [can include parameter, data, and model structure].

*Please provide sensitivity analyses to identify which parameter values are most important in the model. Uncertainty estimates seek to derive a range of credible results on the basis of an exploration of the range of reasonable parameter values. The choice of method should be presented and justified.

*Please discuss the scientific rationale for the choice of model structure and identify points where this choice could influence conclusions drawn. Please also describe the strength of the scientific basis underlying the key model assumptions.

*For studies that develop a prediction model or evaluate its performance, please ensure that the study is reported according to the TRIPOD statement (https://www.equator-network.org/reporting-guidelines/tripod-statement) and include the completed checklist as Supporting Information. Please add the following statement, or similar, to the Methods: "This study is reported as per the Transparent Reporting of a Multivariable Prediction Model for Individual Prognosis Or Diagnosis (TRIPOD) statement (S1 Checklist)." For studies using machine learning, please use the TRIPOD-AI checklist. When completing the checklist, please use section and paragraph numbers, rather than page numbers.

# Author comment: Author response to Decision Letter 2

**DOI:** 10.1371/journal.pmed.1004935.r003

17 Mar 2026

Attachment Submitted filename: Response2-PLOSMED.docx

# Peer review: Decision Letter 2

**DOI:** 10.1371/journal.pmed.1004935.r004

**Authors:** Katarzyna Starowicz

3 Jun 2026

Dear Dr Dubucs,

Many thanks for submitting your manuscript "

Risk stratification for significant acute traumatic intracranial hemorrhage in older adults after a ground-level fall" (PMEDICINE-D-26-00283R2) to PLOS Medicine. The paper has been reviewed by subject experts and a statistician; their comments are included below and can also be accessed here: [LINK]

Please accept my sincere apologies for delay regarding decision. Your manuscript has been seen by all original reviewers. As you will see, the reviewers has some minor additional comments and requests regarding your manuscript. After discussing the paper with the editorial team and an academic editor with relevant expertise, I'm pleased to invite you to revise the paper in response to the reviewers' comments and editorial requests.

In addition to these revisions, you may need to complete some formatting changes, which you will receive in a follow up email. A member of our team will be in touch with a set of requests shortly. If you do not receive a separate email within a few days, please assume that checks have been completed, and no additional changes are required.

When you upload your revision, please include a point-by-point response that addresses all of the reviewer and editorial points, indicating the changes made in the manuscript and either an excerpt of the revised text or the location (eg: page and line number) where each change can be found. You must provide point-by-point response to each of them. Not providing this in form of rebuttal will significantly delay your manuscript.

It may also be helpful to check the guidelines for revised papers at http://journals.plos.org/plosmedicine/s/revising-your-manuscript for any that apply to your paper.

We ask that you submit your revision by Jun 17 2026 11:59PM. However, if this deadline is not feasible, please contact me by email, and we can discuss a suitable alternative.

Don't hesitate to contact me directly with any questions (kstarowicz@plos.org).

Best regards,

Katarzyna

Katarzyna Starowicz, PhD

Associate Editor

PLOS Medicine

kstarowicz@plos.org

-----------------------------------------------------------

Comments from the reviewers:

Reviewer #1: Recommendation: Accept after minor revisions

This revised manuscript is substantially improved and addresses the prior reviewer concerns adequately. The study is clinically important, prospective, multicentre, and focused on a high-volume population in which CT overuse is a relevant problem. The derived PIWI rules are clearly presented and show 100% sensitivity with potential CT reductions of 41.7% and 24.2%, respectively, although external validation remains necessary before clinical implementation.

Minor points to address before acceptance:

*The abstract still contains a residual wording error in the highlighted version: "following a ground-level fall and who underwent a computed tomography scan, such injury broadly." This phrase should be deleted.

*Table 4 contains a typographical error: "95% Conficence Interval" should be "95% Confidence Interval."

*The conclusion should more strongly state that the rules are not yet ready for implementation without independent external validation.

*The Author Summary should include the main limitations in plain language, particularly the absence of external validation and the relatively small number of outcome events.

Overall, the manuscript is suitable for publication once these minor editorial and clarity issues are corrected.

Reviewer #2: This manuscript is a revision of a previous submission. The authors have addressed most reviewer comments appropriately. I am, however, not happy with their response to my (Reviewer 2) previous comment # 5. The authors have now deleted the sentence which I queried " Among those with significant acute intracranial haemorrhage, 49 patients (68.1%) required hospitalization". This would appear "an easy way out", and may be perceived as selective reporting. I suggest this sentence is re-inserted. Perhaps you could add a line stating something like (if correct) "Those not requiring admission to hospital received appropriate observation in the ER for longer periods of time, and were often discharged only after a second CT scan showing no progression". Might you know how many received a second CT Scan?

Minor comments:

1.At various places in the manuscript and in the tables you report on "Reduced GCS from baseline" How did you define "baseline" Was this the presumed pre-injury GCS or the GCS on presentation?

2.Please check the formatting of the References, in particular to the format of listing 6 authors et al.

Reviewer #3: Thanks authors for their effort to clarify my concerns. It's mostly fine as a risk stratification rule (or rules). I am satisfied with the response and revision. No further issues needing attention.

EDITORIAL REQUESTS:

* Please confirm that your title complies with PLOS Medicine's style. Your title must be nondeclarative and not a question. It should begin with main concept if possible. "Effect of" should be used only if causality can be inferred, i.e., for an RCT. Please place the study design ("A randomized controlled trial," "A retrospective study," "A modelling study," etc.) in the subtitle (ie, after a colon).

* Please confirm that your abstract complies with our requirements, including format (three sections: Background, Methods and Findings, and Conclusions) and providing all the information relevant to this study type https://journals.plos.org/plosmedicine/s/submission-guidelines#loc-abstract

* Please ensure that the Introduction ends with a clear description of the study question or hypothesis.

* Please ensure that all abbreviations are defined at first use throughout the text.

* Please confirm that all numbers presented in the abstract are present and identical to numbers presented in the main manuscript text.

* Please disclose any use of AI tools and technologies to the study or article content at the end of the Methods. Please specify the name(s) and version(s) of any tool(s) used and a description of their use, including how the AI-assisted or generated content was evaluated by authors. Please also include a statement specifying who retains responsibility for the final manuscript. Please see our guidelines for more information: https://journals.plos.org/plosmedicine/s/ethical-publishing-practice#loc-artificial-intelligence-tools-and-technologies.

* Please review your text for claims of novelty or primacy (e.g. 'for the first time') and remove this language. In addition, please check that any use of statistical terms (such as trend or significant) are supported by the data, and if not please remove them.

* Please remove the 'conclusions' subheading from the discussion. Please also remove any other subheadings from the discussion.

* Statistical reporting: Please revise throughout the manuscript, including tables and figures.

- Please report statistical information as follows to improve clarity for the reader ""22% (95% CI [13,28]; p</=)"".

- Please separate upper and lower bounds with commas instead of hyphens as the latter can be confused with reporting of negative values.

- Please repeat statistical definitions (HR, CI etc.) for each set of parentheses.

* In the last sentence of the Abstract Methods and Findings section, please describe the main limitation(s) of the study's methodology.

* In the abstract, please include the important dependent variables that are adjusted for in the analyses.

* In the author summary, please revise formatting and ensure you use bullet points.

* In the author summary, in the final bullet point of 'What Do These Findings Mean?', please include the main limitations of the study in non-technical language.

* The funding statement should include: specific grant numbers, initials of authors who received each award, URLs to sponsors’ websites. Also, please state whether any sponsors or funders (other than the named authors) played any role in study design, data collection and analysis, the decision to publish, or preparation of the manuscript. If they had no role in the research, include this sentence: “The funders had no role in study design, data collection and analysis,

decision to publish, or preparation of the manuscript.”

* PLOS Medicine requires that the de-identified data underlying the specific results in a published article be made available, without restrictions on access, in a public repository or as Supporting Information at the time of article publication, provided it is legal and ethical to do so. Please see the policy at

http://journals.plos.org/plosmedicine/s/data-availability and FAQs at http://journals.plos.org/plosmedicine/s/data-availability#loc-faqs-for-data-policy "

* PLOS defines the “minimal data set” to consist of the data set used to reach the conclusions drawn in the manuscript with related metadata and methods, and any additional data required to replicate the reported study findings in their entirety. Authors do not need to submit their entire data set, or the raw data collected during an investigation. Please submit the following data:

The values behind the means, standard deviations and other measures reported;

The values used to build graphs;

The points extracted from images for analysis."

* The Data Availability Statement (DAS) requires revision. For each data source used in your study:

a) If the data are freely or publicly available, note this and state the location of the data: within the paper, in Supporting Information files, or in a public repository (include the DOI or accession number).

b) If the data are owned by a third party but freely available upon request, please note this and state the owner of the data set and contact information for data requests (web or email address). Note that a study author cannot be the contact person for the data.

c) If the data are not freely available, please describe briefly the ethical, legal, or contractual restriction that prevents you from sharing it. Please also include an appropriate contact (web or email address) for inquiries (again, this cannot be a study author).

* For studies in which a novel model is central to the manuscript's findings, as is the case here, authors are responsible for providing the source code needed to replicate the study's findings in a repository (such as GitHub, SourceForge or Bitbucket) or a cloud computing service (such as Code Ocean). Please include a citable digital object identifier (DOI) or other persistent identifier of the code (such as through linkage with Zenodo). Protection of authors’ intellectual property will not be cause for exception. Please explain in the manuscript’s Data Availability Statement how readers can access the shared code.

* Thank you for agreeing to make your data available. At this time, please provide the link to the data repository and accession numbers required for access.

* Please include the statement on code availability in the data availability statement. Please see guidance here:https://journals.plos.org/plosmedicine/s/code-sharing-guidance#loc-data-availability-statement-examples

* Please provide the name(s) of the institutional review board(s) that provided ethical approval.

* Please revise for use of patient-centered language. Please note that patient-centered language is constructed with the use of post-modified nouns (e.g. 'patients with psoriasis’ (or similar) instead of ‘psoriasis patients’) putting the person first in the sentence structure.

* Please specify whether informed consent was written or oral. Please ensure that the research complies with the PLOS policy in full: https://journals.plos.org/plosmedicine/s/human-subjects-research#loc-patient-privacy-and-informed-consent-for-publication

* Please provide titles and legends for all figures and tables (including those in Supporting Information files). Please define all acronyms used in each figure or table in its corresponding legend.

* When a p value is given, please specify the statistical test used to determine it in the legend.

* Please consider avoiding the use of red and green in order to make your figure more accessible

* Please provide the unadjusted comparisons as well as the adjusted comparisons in all relevant Tables

* Please specify the variables controlled for in all relevant Tables

SUPPORTING INFORMATION:

* We have noticed that you have uploaded a list of legend in Supporting Information file. Please add a full list of legends for your Supporting Information files after the references list.

* We do recommend upload each supporting information file separately. You may use almost any description as the item name of your supporting information as long as it contains an "S" and number. For example, “S1 Appendix” and “S2 Appendix,” “S1 Table” and “S2 Table,” and so forth.

* Use whole numbers when naming your supporting information files. Combine separate parts (e.g., S1A and S1B Table) into one file (e.g. S1 Table) or rename with whole numbers (e.g., S1 and S2 Table).

* Match the names of your supporting information files with the supporting information captions within your manuscript. For example, a PDF file for “S2 Fig.” must be named “S2_fig.pdf”.

* The supporting information name and number are required in a caption, and we highly recommend including a one-line title as well. You may also include a legend in your caption, but it is not required. Format your supporting information captions as follows:

S1 Text. Title is strongly recommended. Legend is optional.

* In the published article, supporting information files are accessed only through a hyperlink attached to the captions. For this reason, you must list captions at the end of your manuscript file. You may include a caption within the supporting information file itself, as long as that caption is also provided in the manuscript file. Do not submit a separate caption file.

* We do recommend that STROBE and TRIPOD Checklists are listed as S1 Checklist, S2 Checklist respectively.

* Please revise your references to supporting information in the manuscript main text after implementing changes

* Please include IRB approval as "Other" type of document in your resubmission as this document is for editorial purposes only

* Please include The Study Protocol in English

# Author comment: Author response to Decision Letter 3

**DOI:** 10.1371/journal.pmed.1004935.r005

16 Jun 2026

Attachment Submitted filename: Response.docx

# Peer review: Decision Letter 3

**DOI:** 10.1371/journal.pmed.1004935.r006

**Authors:** Katarzyna Starowicz

23 Jun 2026

Dear Dr Dubucs,

On behalf of Dr. Starowicz, our colleagues, and the Academic Editor, Dr. Martin Schreiber, I am pleased to inform you that we have agreed to publish your manuscript "Risk stratification for significant acute traumatic intracranial hemorrhage in older adults after a ground-level fall: A prospective multicentre cohort study" (PMEDICINE-D-26-00283R3) in PLOS Medicine.

Before your manuscript can be formally accepted you will need to complete some formatting changes, which you will receive in a follow up email. Please be aware that it may take several days for you to receive this email; during this time no action is required by you. Once you have received these formatting requests, please note that your manuscript will not be scheduled for publication until you have made the required changes.

Please also note the following requests:

Please remove 'reasonable' from the Data Availability statement.

Please remove '&' from the subscripts in the author list (multiple authors) and/or correct.

Please clarify why the corresponding author has the subscript '¶*' in the author list.

Lines 52-53: Please rearrange phrasing to use active voice, as follows: 'We designed two highly sensitive clinical decision rules, both based on simple and reliable criteria, specifically for application to this patient population."

Line 57, insert 'to our knowledge' to read, "Our study provides a, to our knowledge, novel approach" or delete novel and use a different qualifier (avoiding claims of primacy).

Line 285: hemorrhage should be plural.

Figure 2, bottom right: a closed bracket is missing here: (95% CI [0, 1)

Line 350: revise to "could potentially reduce"

Line 381: correct spelling of ' baselin' to ' baseline'

Line 392: revise to "focused on"

Please confirm to editors whether any AI tools were used in the assembly or analysis of this study and add any appropriate disclosure, as previously indicated by the editor.

In the meantime, please log into Editorial Manager at http://www.editorialmanager.com/pmedicine/, click the "Update My Information" link at the top of the page, and update your user information to ensure an efficient production process.

PRESS

We frequently collaborate with press offices. If your institution or institutions have a press office, please notify them about your upcoming paper at this point, to enable them to help maximise its impact. If the press office is planning to promote your findings, we would be grateful if they could coordinate with medicinepress@plos.org. If you have not yet opted out of the early version process, we ask that you notify us immediately of any press plans so that we may do so on your behalf.

We also ask that you take this opportunity to read our Embargo Policy regarding the discussion, promotion and media coverage of work that is yet to be published by PLOS. As your manuscript is not yet published, it is bound by the conditions of our Embargo Policy. Please be aware that this policy is in place both to ensure that any press coverage of your article is fully substantiated and to provide a direct link between such coverage and the published work. For full details of our Embargo Policy, please visit http://www.plos.org/about/media-inquiries/embargo-policy/.

To enhance the reproducibility of your results, we recommend that you deposit your laboratory protocols in protocols.io, where a protocol can be assigned its own identifier (DOI) such that it can be cited independently in the future. Additionally, PLOS ONE offers an option to publish peer-reviewed clinical study protocols. Read more information on sharing protocols at https://plos.org/protocols?utm_medium=editorial-email&utm_source=authorletters&utm_campaign=protocols

Thank you again for submitting to PLOS Medicine. We look forward to publishing your paper.

Sincerely,

Alison Farrell, PhD

Senior Editor

PLOS Medicine
